# Light Scattering of Leaf Surface and Spongy Mesophyll and Concentration of Anthocyanin Influence Typical and Modified Photochemical Reflectance Indices

**DOI:** 10.3390/plants14213255

**Published:** 2025-10-24

**Authors:** Ekaterina Sukhova, Lyubov Yudina, Yuriy Zolin, Alyona Popova, Kseniya Grebneva, Karina Abasheva, Elizaveta Kozlova, Vladimir Sukhov

**Affiliations:** Department of Biophysics, N.I. Lobachevsky State University of Nizhny Novgorod, 603950 Nizhny Novgorod, Russia; n.catherine@inbox.ru (E.S.); lyubovsurova@mail.ru (L.Y.); uchebnayap.zolin@gmail.com (Y.Z.); silverkumiho@mail.ru (A.P.); grebneva.kseniya01@mail.ru (K.G.); karinarutter@yandex.ru (K.A.); elizagreen21@gmail.com (E.K.)

**Keywords:** photochemical reflectance index (PRI), leaf reflectance model, light scattering, rough surface, spongy mesophyll, anthocyanin, dicot plants

## Abstract

The photochemical reflectance index (PRI) based on reflectance at 531 and 570 nm and its modifications with shifted measuring wavelengths are well-known indicators of stress changes in photosynthetic processes which can be induced in plants under the action of numerous adverse factors (e.g., drought). However, the relationships between photosynthetic characteristics and the PRI are varied in different works; this means that photosynthetic responses are not the only reason for PRI changes. In the current work, we analyzed the influence of the light scattering of leaf surfaces and mesophyll layers and concentrations of leaf pigments on typical and modified PRIs. The analytical model of light reflectance and transmittance in the leaves of dicot plants, which had been previously developed in our work, and experimental measurements were used to analyze this influence. It was shown that increasing the light scattering of the leaf surface and the anthocyanin concentration and decreasing the light scattering of the spongy mesophyll increased PRIs with short measuring wavelengths and decreased PRIs with long measuring wavelengths. The action of drought induced similar changes in typical and modified PRIs, which were accompanied by an increased light scattering of the leaf surface and anthocyanin concentration and a decreased light scattering of the spongy mesophyll. The results show that changes in the light scattering of the leaf surface and spongy mesophyll and in the anthocyanin concentration can be the important mechanisms of slow changes in typical and modified PRIs, including drought-induced ones.

## 1. Introduction

Terrestrial plants can be affected by the action of numerous abiotic stressors (drought [1,2], high [3,4] and low [5] temperatures, high-intensity light [6], excess precipitation [7], and others) which inhibit their physiological processes. Photosynthesis, which is the basis of plant productivity, is an important target of abiotic stressors; particularly, it can be disrupted under the action of drought [8,9,10], heat [11,12], or excess light [13,14]. From a practical point of view, this means that the early detection of stressor-induced photosynthetic changes is an important problem for the remote and proximal sensing of plants because this detection can be the basis for the timely use of methods of plant protection.

There are varying methods for the remote and proximal sensing of stressor actions on plants [15,16]; particularly, an analysis of the spectral characteristics of reflected light is an effective tool to detect plant changes under the action of adverse factors [15,17,18]. It is known that reflectance spectra can be sensitive to changes in the concentration of photosynthetic pigments [19,20,21,22], water [23,24] and nitrogen [25,26] content, leaf area index and biomass [18,27,28,29], etc. An analysis of dimensionless reflectance indices, which are mainly calculated on the basis of reflectance at two or three wavelengths, can additionally increase the efficiency of plant monitoring [15,16,18].

It was shown [30,31] that light reflectance at about 531 nm (R_531_) is decreased under the action of the excess light for minutes; in contrast, changes in the reflectance at 570 nm (R_570_) are weak. Based on these results, the photochemical reflectance index (PRI) was proposed [31,32,33]. The typical PRI can be calculated in accordance with Equation (1) [33,34,35,36]:

(1) $$\mathrm{PRI}=\frac{R_{531}-R_{570}}{R_{531}+R_{570}},$$

Numerous works show that the PRI can be changed under the action of varying stressors, including excess light [31,37], drought [38,39,40,41], non-optimal temperatures [39,41], salinization [42,43], nitrogen deficit [44], and others.

Traditionally, transitions in the xanthophyll cycle, which are induced by the acidification of the chloroplast lumen [14,45,46], are considered as the main mechanism of a quickly decreasing PRI under the action of stressors [31,32,33,47]. The chloroplast shrinkage induced by this acidification is considered to be an additional mechanism of this fast decreasing [35,37]. The lumen acidification, which can directly induce a decreasing PRI [47], is an important plant response on the action of stressors [13,14]. It is strongly related to photosynthetic processes through the induction of the non-photochemical quenching of fluorescence [14,46] and the suppression of photosynthetic linear electron flow [48,49]. As a result, it can be expected that changes in the typical PRI should be strongly related to changes in photosynthetic parameters.

There are works [31,32,33,37,50,51] showing strong relationships between the PRI and photosynthetic parameters (mainly, parameters of photosynthetic light reactions); moreover, the inhibition of photosynthetic activity by dichlorophenyl dimethylurea suppresses light-induced changes in the PRI [52]. However, our meta-analysis [36] shows that linear correlation coefficients between the PRI and photosynthetic parameters can be strongly varied and dependent on the conditions of measurements. Recent experimental works also support that relationships between the PRI and photosynthetic parameters can be moderate or weak [52,53,54]; particularly, these relationships are weak in photoinhibited leaves [53,54]. Additionally, our results [41,55] show that the typical PRI can be increased or decreased under the long-term action of stressors (e.g., drought) in different variants of the experiment; as a result, the correlation between the typical PRI and specific photosynthetic parameters can be positive or negative in different cases.

Potentially, varying relationships between the PRI and photosynthetic parameters can be caused by additional mechanisms of changes in the photochemical reflectance index. It is known [56,57,58,59] that changes in the PRI can be related to changes in concentrations of chlorophylls and carotenoids and in ratios between these concentrations. These mechanisms participate in forming the seasonal dynamics of the PRI [59]; however, it cannot be excluded that changes in the photosynthetic pigment content can also be formed under the action of long-term stressors (e.g., under drought) and modify the PRI in these cases [55]. Wong and Gamon [58] proposed additional mechanisms of long-term changes in the PRI under the action of deep cold: decreasing the total reflectance through, probably, a change in internal light scattering.

There are several potential ways to minimize the influence of noted factors: to use light-induced changes in the PRI [47,52,55,60,61,62], to analyze the spatial heterogeneity of PRI distribution [63], and to measure modified PRIs [41,43,64,65]. In accordance with the last method [64], modified PRIs (PRI(λ,570)) can be calculated on the basis of short or long measuring wavelengths λ:

(2) $$\mathrm{PRI}\left(\lambda ,570\right)=\frac{R_{\lambda }-R_{570}}{R_{\lambda }+R_{570}},$$

where R_λ_ is the reflectance at λ. Using PRI(λ,570) with short and long measuring wavelengths is based on two components of fast PRI changes (fast-relaxing and slow-relaxing ones), which were shown early on [33,64]. Previously, we showed that typical (λ = 531) and modified (λ = 505, 515, 525, 535, 545, and 555 nm) PRIs are sensitive to the action of light [64], high temperature [41], drought [41], and salinization [43]. All PRI(λ,570) are decreased under the short-term action of excess light [64]. In contrast, the action of long-term stressors (1 day and more), including drought [41] and salinization [43], positively shifts PRI(λ,570) with λ < about 531 nm and negatively shifts PRI(λ,570) with λ > about 531 nm. The direction of changes in the typical PRI can be varied in different experiments (e.g., PRI(531,570) can be increased or decreased under the action of water deficit on pea plants [41,55]). The results show that the mechanisms of slow changes in PRI(λ,570) under the action of long-term stressors are different from the mechanisms of fast changes in these indices under the action of excess light and, maybe, other short-term stressors.

Earlier, we hypothesized [41,43] that changes in typical and modified PRIs under the action of long-term stressors can be, potentially, caused by shifts in the concentrations of chlorophylls a and b and carotenoids. However, the spectra of light absorption of these photosynthetic pigments in the 500–600 nm spectral region [66] show that changes in the concentrations of chlorophylls a and b and carotenoids should influence typical and modified PRIs in an intricate manner: changes the in concentration of carotenoids should strongly influence PRI(λ,570) with a short λ and weakly influence PRI(λ,570) with a long λ; in contrast, changes in chlorophylls a and b should similarly influence all typical and modified PRIs through changes in R_570_, which is the reference wavelength for these PRIs. However, experimental results [41,43] show that long-term stressors induce opposite changes in PRI(λ,570) with short and long measuring wavelengths; the results do not seem to be in accordance with changes in both concentrations of chlorophylls and carotenoids.

Considering these points, we hypothesize that changes in typical and modified PRIs can be related to changes in light scattering, since these changes modify leaf reflectance spectra [67] and can influence the typical PRI under deep cold action [58]. The light scattering can include different components [67]: the light scattering of the leaf surface (in the rough surface), high light scattering of the spongy mesophyll, and weak light scattering of the palisade mesophyll; the last component is minor.

Thus, our current work was mainly focused on an analysis of the role of changes in the light scattering of leaf surfaces and mesophyll layers in the slow changes of typical and modified PRIs. Additionally, we also analyzed the influence of changes in the concentrations of photosynthetic pigments (chlorophylls a and b, carotenoids, and anthocyanin) on these PRIs to verify the earlier hypothesis about influencing these changes in typical and modified PRIs. The previously developed mathematical model of light reflectance and transmittance in the leaves of dicot plants (with palisade and spongy mesophyll layers) [67] was used to analyze this problem. This model could describe light scattering by the leaf surface and mesophyll layers and light absorption by photosynthetic pigments.

## 2. Analytical Model of Light Reflectance and Transmittance in Leaves of Dicot Plants

### 2.1. Brief Description of the Analytical Model of Light Reflectance and Transmittance in Leaves

**Figure 1:**
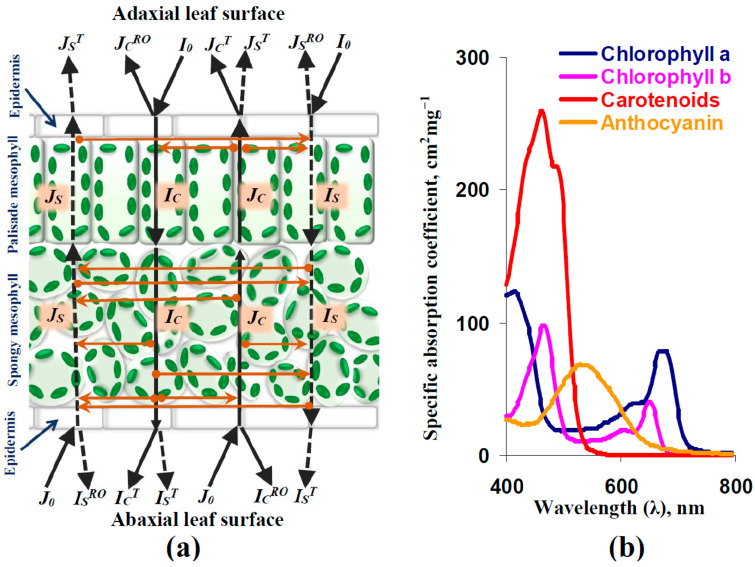
(**a**) Scheme of the analytical model of light reflectance and transmittance in leaves of dicot plants (on the basis of [67]). Forward and backward light flows are shown by black solid and dotted lines. Transformations between light flows are shown by orange lines. The model describes intensities of the forward-collimated (*I_C_*) and scattered (*I_S_*) light, backward-collimated (*J_C_*) and scattered (*J_S_*) light, forward-(*I*_0_) and backward-(*J*_0_) collimated light directed to adaxial and abaxial surfaces of leaves (the incident light), collimated light reflecting out from adaxial (*J_C_^RO^*) and abaxial (*I_C_^RO^*) leaf surfaces in air, scattered light reflecting out from adaxial (*J_S_^RO^*) and abaxial (*I_S_^RO^*) leaf surfaces in air, collimated light transferring from leaves to air through adaxial (*J_C_^T^*) and abaxial (*I_C_^T^*) surfaces, and scattered light transferring from leaves to air through adaxial (*J_S_^T^*) and abaxial (*I_S_^T^*) surfaces. Model equations and parameters are described in detail in our previous work [67] and File S1 (Supplementary Materials). (**b**) Spectra of coefficients of specific light absorption for photosynthetic pigments. Spectra of chlorophyll a, chlorophyll b, carotenoids (using the example of β-carotene), and anthocyanin are shown. They were constructed on the basis of [66,67].

Our analytical model of light reflectance and transmittance in the leaves of dicot plants, which have both palisade and spongy mesophyll (Figure 1a), was described in the previous work [67] in detail. Equations for the model are shown in File S1 (Supplementary Materials).

Briefly, this model described four variables, including the intensities of forward-collimated light (*I_C_*), backward-collimated light (*J_C_*), forward-scattered light (*I_S_*), and backward-scattered light (*J_S_*). The following processes were analyzed in the model:

- Light reflectance, transmittance, and scattering of adaxial and abaxial leaf surfaces: Snell’s and Fresnel’s laws were used as the basis of their descriptions [68,69]. It was considered that some fraction of the leaf surface was rough and scattered the collimated light.
- Light transmittance and scattering of the palisade mesophyll layer: It was assumed that light scattering in this layer was low (in accordance with [70]). As a result, this scattering was not considered in the main description of light flows in the palisade mesophyll; the Beer–Bouguer–Lambert law was used for this description [71]. However, light scattering of the palisade mesophyll was described as the additional component of reflectance in the adaxial leaf surface (see Equation (S38) in File S1).
- Light transmittance and scattering of the spongy mesophyll layer: It is known that this layer has a high coefficient of light scattering [70]. As a result, optical properties of the spongy mesophyll layer were described on the basis of the Kubelka–Munk model, which considered four light flows including forward- and backward-collimated light and forward- and backward-scattered light [72,73].

The light absorption spectrum was described as the sum of the products of the concentrations of photosynthetic pigments and the spectra of their specific light absorption coefficients (see Equation (S61) in File S1). It should be noted that anthocyanin was not described in the original model [67]; in contrast, it was included in the current variant of the model. We assumed that the basic concentration of anthocyanin was zero because the production of this pigment is stimulated by the action of abiotic stressors (including, e.g., drought) [74,75,76]. Figure 1b shows the spectra of the specific light absorption coefficients of chlorophyll a, chlorophyll b, carotenoids, and anthocyanin, which were used in our model.

Finally, it should be noted that our model did not describe light-induced fast changes in light absorption with maximums at about 526 and 545 nm [33], which are possibly the mechanisms of fast changes in typical and modified PRIs [64]. The current model described only the changes in light reflectance induced by the changes in light scattering and absorption, which should be results of the relatively slow processes (e.g., changes in the fraction of the rough surface or concentrations of photosynthetic pigments).

### 2.2. Parameters of the Analytical Model of Light Reflectance and Transmittance in Leaves

The basic values of the parameters for the analytical model of light reflectance and transmittance in leaves were mainly estimated in our previous work [67] (for pea leaves) and are included in Table 1. Anthocyanins were not considered in [67]; as a result, we used zero as the basic value of the average anthocyanin concentration.

We mainly used the values of parameters which were decreased by 50% or increased by 50% to analyze the influence of their changes on typical and modified photochemical reflectance indices. To investigate the influence of anthocyanins, we used only increased anthocyanin concentrations (non-zero concentrations).

## 3. Results

### 3.1. Typical and Modified Photochemical Reflectance Indices Described by the Model at Basic Values of Parameters

**Figure 2:**
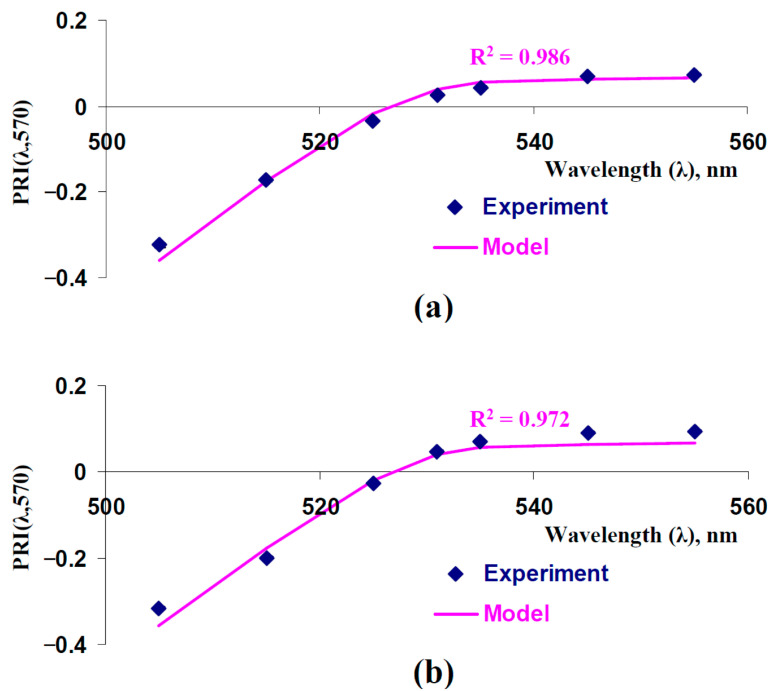
Measured and model-based dependences of typical and modified photochemical reflectance indices (PRI(λ,570)) on the measuring wavelength (λ). Model-based dependence was calculated using basic parameters of the model of light reflectance and transmittance in plant leaves (Table 1). (**a**) Experimental results of our previous work [43] are shown (average control values of the PRI(λ,570)). A hyperspectral camera was used to measure reflectance spectra in peas. (**b**) Experimental results, which were measured in the current work, are shown. Handheld PolyPen RP 410 UVIS system (Photon Systems Instruments, Drásov, Czech) was used to measure reflectance spectra of leaves (*n* = 312). Equation (2), where R_λ_ is reflectance at the measuring wavelength (λ) and R_570_ is reflectance at the reference wavelength (570 nm), was used to calculate typical and modified photochemical reflectance indices. In accordance with [43], λ was 505, 515, 525, 531, 535, 545, and 555 nm. R^2^ is the determination coefficient for experimental and model-based dependences.

In the first step of the current investigation, we analyzed typical and modified PRIs which were described by the model and were shown in the experiments (Figure 2). Figure 2a shows the experimental dependence of PRI[λ,570] on λ from our previous work [43]; in this case, PRIs were measured using the hyperspectral camera in the leaves of control peas (with irrigation). Figure 2b shows the results of the current work, which were measured using the handheld PolyPen RP 410 UVIS system in the leaves of control peas. Both experimental dependences were similar to the model-based dependence (coefficients of determination were more than 0.97), which was calculated at the basic model parameters (see Table 1).

It was shown that the developed model accurately described typical and modified PRIs and, therefore, could be used for further analysis of the changes in these photochemical reflectance indices.

### 3.2. Influence of Light Scattering of Leaf Surface and Mesophyll Layers on Typical and Modified Photochemical Reflectance Indices

**Figure 3:**
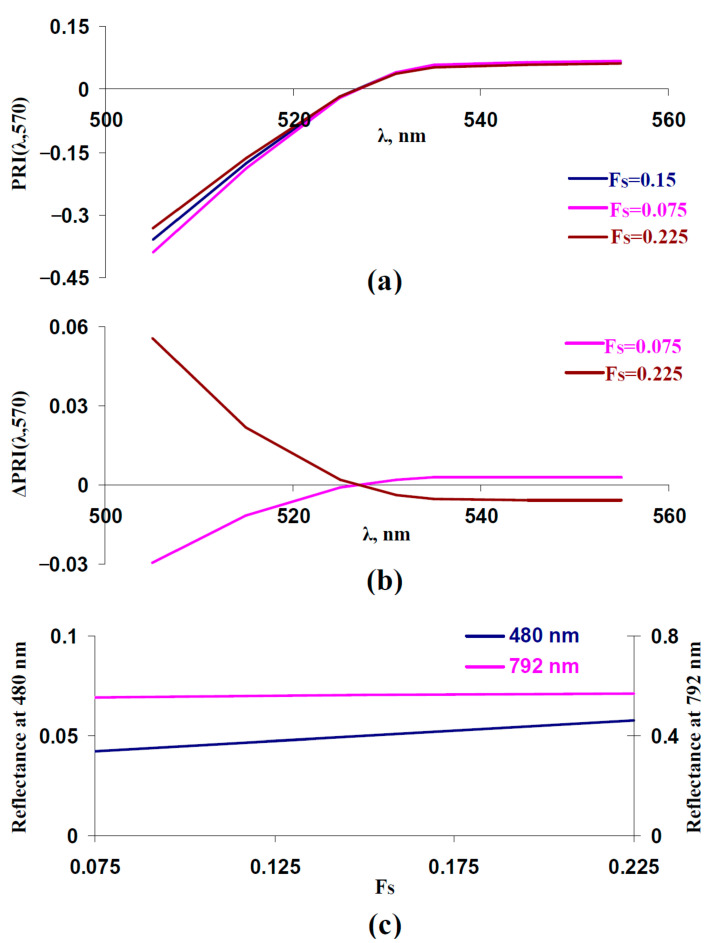
Influence of fraction of the rough surface (F_S_) on PRI(λ,570) and reflectance at 480 and 792 nm. Results of model-based calculation are shown. (**a**) Model-based dependences of PRI(λ,570) on λ, which were calculated at F_S_ = 0.15 (basic value), F_S_ = 0.075 (low value), and F_S_ = 0.225 (high value). Other parameters of the model of light reflectance and transmittance in plant leaves had basic values (Table 1). (**b**) Dependences of changes in PRI(λ,570) (ΔPRI(λ,570)) on λ. ΔPRI(λ,570) were calculated as the difference between PRI(λ,570) at F_S_ = 0.075 or F_S_ = 0.225 and PRI(λ,570) at F_S_ = 0.15. (**c**) Dependences of reflectance at 480 and 792 nm on F_S_.

In the next stage of the current work, we investigated the influence of the fraction of the rough surface (F_S_) on typical and modified PRIs. It was shown (Figure 3a,b) that increasing F_S_ increased PRI(λ,570) at λ < 531 nm and decreased PRI(λ,570) at λ ≥ 531 nm; this dependence was in a good accordance with the experimental results, which were shown under the action of drought [41], salinization [43], and heating [41]. In contrast, decreasing F_S_ increased PRI(λ,570) at λ ≥ 531 nm and decreased PRI(λ,570) at λ < 531 nm. As a result, we supposed that increasing F_S_ could be a potential way to slow changes in typical and modified PRIs under the long-term action of stressors.

Figure 3c shows that increasing F_S_ weakly influenced the reflectance at 792 nm; in contrast, this increase strongly increased the reflectance at 480 nm. This result showed that the reflectance at 480 nm could be used to estimate F_S_. This was in a good accordance with the results of a previous analysis of the model [67], which showed that changes in F_S_ strongly influenced reflectance in the approximately 400–500 nm spectral region; the reflectance in other spectral regions was weakly dependent on F_S_.

**Figure 4:**
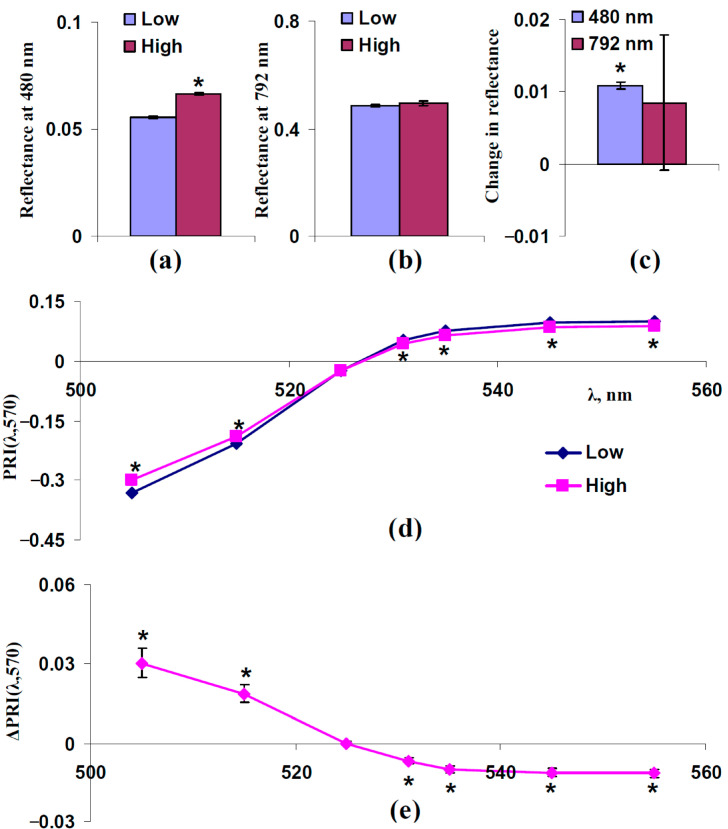
Experimental analysis of influence of F_S_ on PRI(λ,570). 312 measurements of reflectance spectra in pea plants were ranged in accordance with values of reflectance at 480 nm, since this reflectance is mainly related to F_S_ (Figure 3), and were divided into two equal groups (*n* = 156): “Low” and “High”. (**a**) Reflectance at 480 nm in these groups. (**b**) Reflectance at 792 nm in these groups. (**c**) Changes in reflectance at 480 and 792 nm, which were calculated as the difference between ones in the “High” and “Low” groups. (**d**) Dependences of PRI(λ,570) on λ in “Low” and “High” groups. (**e**) Dependence of changes in PRI(λ,570) (ΔPRI(λ,570)) on λ. ΔPRI(λ,570) were calculated as the difference between PRI(λ,570) in the “High” group and PRI(λ,570) in the “Low” group. *, differences between groups are significant (*p* < 0.05).

Figure 4 shows the experimental influence of F_S_, which was estimated on the basis of reflectance at 480 nm, on typical and modified PRIs in the leaves of control pea plants. For analysis, individual experimental results were divided into two equal groups: “Low”, which includes plants with a reflectance 480 nm lower than the median of this reflectance, and “High”, which includes plants with a reflectance 480 nm higher than the median of this reflectance. The median was calculated on the basis of all plants under irrigation. The reflectance at 480 and 792 nm and typical and modified PRIs in these groups were compared.

It was shown that the reflectance at 480 nm was significantly different in these groups (Figure 4a,c); in contrast, significant differences in the reflectance at 792 nm were absent (Figure 4a,c). The last results showed that light scattering of the spongy mesophyll was not different in the investigated groups.

The analysis of typical and modified PRIs showed that increasing the reflectance at 480 nm (increasing F_S_) increased PRI(λ,570) at λ < 531 nm and decreased PRI(λ,570) at λ ≥ 531 nm (Figure 4d,e). The result is in good accordance with the results of the model-based analysis (Figure 3).

**Figure 5:**
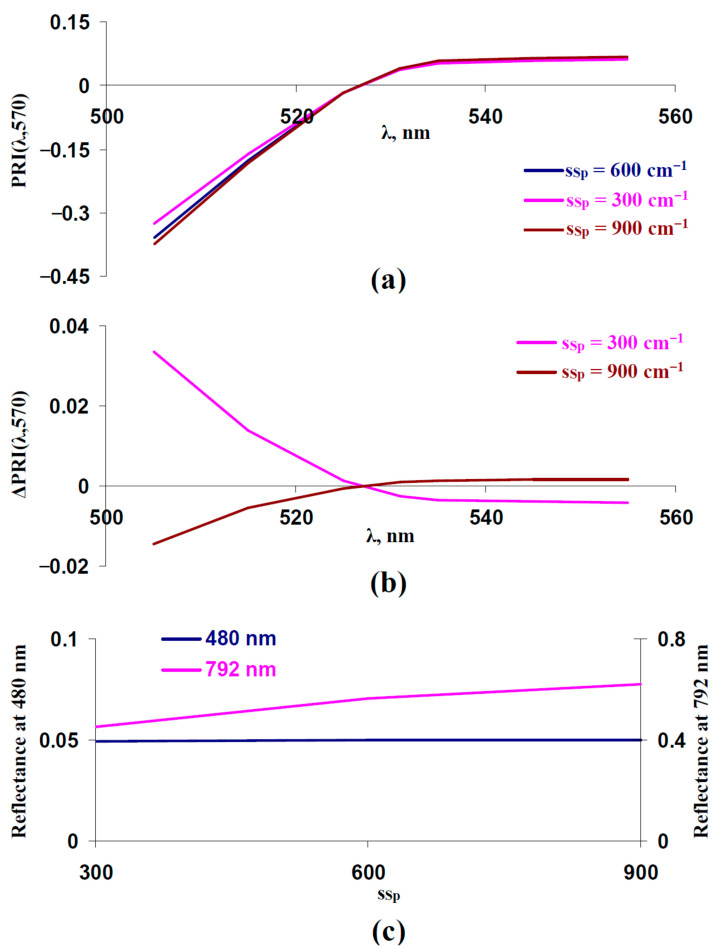
Influence of the light scattering coefficient in the spongy mesophyll layer (s_Sp_) on PRI(λ,570) and reflectance at 480 and 792 nm. Results of model-based calculation are shown. (**a**) Model-based dependences of PRI(λ,570) on λ, which were calculated at s_Sp_ = 600 cm^−1^ (basic value), s_Sp_ = 300 cm^−1^ (low value), and s_Sp_ = 900 cm^−1^ (high value). Other parameters of the model of light reflectance and transmittance in plant leaf were basic (Table 1). (**b**) Dependences of changes in PRI(λ,570) (ΔPRI(λ,570)) on λ. ΔPRI(λ,570) were calculated as the difference between PRI(λ,570) at s_Sp_ = 300 cm^−1^ or s_Sp_ = 900 cm^−1^ and PRI(λ,570) at s_Sp_ = 600 cm^−1^. (**c**) Dependences of reflectance at 480 and 792 nm on s_Sp_.

After that, we investigated the influence of the light scattering coefficient in the spongy mesophyll layer (s_Sp_) on typical and modified PRIs. It was shown (Figure 5a,b) that decreasing the s_Sp_ increased PRI(λ,570) at λ < 531 nm and decreased PRI(λ,570) at λ ≥ 531 nm; this dependence was in good accordance with the experimental results, which were shown under the action of drought [41], salinization [43], and heating [41]. In contrast, increasing the s_Sp_ increased PRI(λ,570) at λ ≥ 531 nm and decreased PRI(λ,570) at λ < 531 nm. As a result, we supposed that decreasing the s_Sp_ could be another potential way of slowing changes in typical and modified PRIs under the long-term action of stressors.

Figure 5c shows that decreasing the s_Sp_ weakly influenced reflectance at 480 nm; in contrast, this decrease strongly decreased reflectance at 792 nm. As a result, the reflectance at 792 nm could be used to estimate s_Sp_. This was in good accordance with the results of previous analyses of the model [67], which showed that changes in s_Sp_ strongly influenced reflectance in the approximately 750–800 nm spectral region; the reflectance in other spectral regions was weakly dependent on s_Sp_.

**Figure 6:**
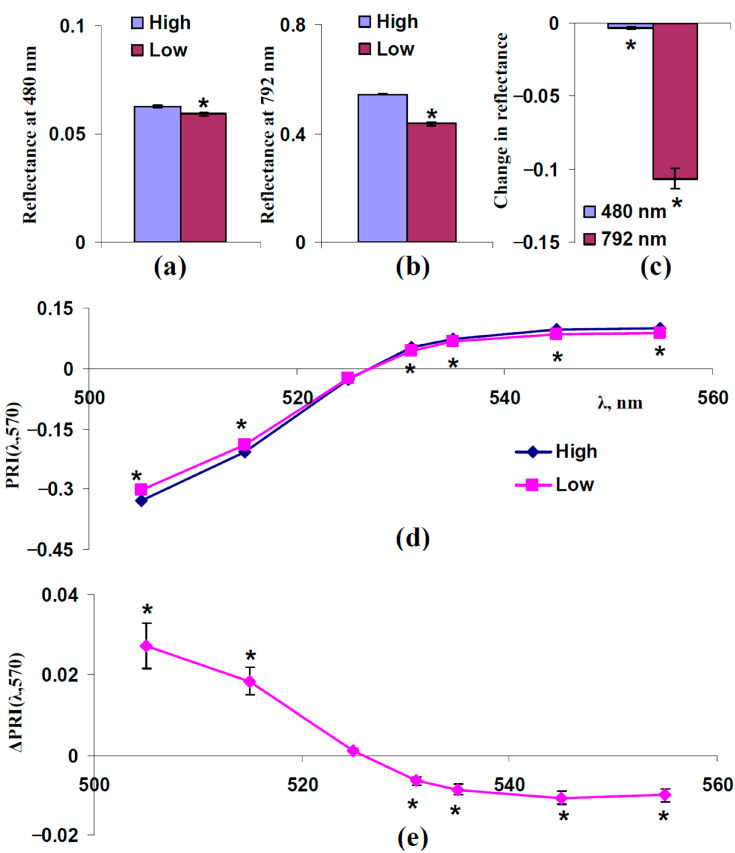
Experimental analysis of influence of s_Sp_ on PRI(λ,570). The 312 measurements of reflectance spectra in pea plants were ranged in accordance with values of reflectance at 792 nm, since this reflectance is mainly related to s_Sp_ (Figure 5), and were divided into two equal groups (*n* = 156): “Low” and “High”. (**a**) Reflectance at 480 nm in these groups. (**b**) Reflectance at 792 nm in these groups. (**c**) Changes in reflectance at 480 and 792 nm, which were calculated as the difference between ones in “Low” and “High” groups. (**d**) Dependences of PRI(λ,570) on λ in “High” and “Low” groups. (**e**) Dependence of changes in PRI(λ,570) (ΔPRI(λ,570)) on λ. ΔPRI(λ,570) were calculated as the difference between PRI(λ,570) in the “Low” group and PRI(λ,570) in the “High” group. *, differences between groups are significant (*p* < 0.05).

Figure 6 shows the experimental influence of s_Sp_, which was estimated on the basis of the reflectance at 792 nm, on typical and modified PRIs in the leaves of control pea plants. For analysis, individual experimental results were divided into two equal groups: “Low”, which includes plants with a reflectance 792 nm lower than the median of this reflectance, and “High”, which includes plants with a reflectance 792 nm higher than the median of this reflectance. The median was calculated on the basis of all plants under irrigation. The reflectance at 480 and 792 nm and typical and modified PRIs in these groups were compared.

It was shown that the reflectance at 792 nm was significantly different in these groups (Figure 6b,c). The reflectance at 480 nm in the “High” group was significantly higher than that of the “Low” group (Figure 6a,c); however, this difference was small (about 5%).

The analysis of typical and modified PRIs showed that decreasing the reflectance at 792 nm (decreasing s_Sp_) increased PRI(λ,570) at λ < 531 nm and decreased PRI(λ,570) at λ ≥ 531 nm (Figure 6d,e). The result is in good accordance with the results of the model-based analysis (Figure 5). It should be noted that the small additional decrease in reflectance at 480 nm (decreasing F_S_, Figure 6a,c) should oppositely influence typical and modified PRIs and, therefore, decrease the magnitudes of their changes; i.e., the changes shown in typical and modified PRIs (Figure 6e) were not related to decreasing F_S_.

Finally, the influence of the light scattering coefficient in the palisade mesophyll layer (s_P_) on PRI(λ,570) was also investigated (Figure S1). The model-based analysis showed that increasing s_P_ increased PRI(λ,570) at λ < 531 nm and decreased PRI(λ,570) at λ ≥ 531 nm and vice versa. However, the magnitudes of all these differences were strongly lower than those at changes in F_S_ and s_Sp_.

### 3.3. Analysis of Participation of Changes in Light Scattering of Leaf Surface and Spongy Mesophyll Layer in Changes in Typical and Modified Photochemical Reflectance Indices Under Drought

**Figure 7:**
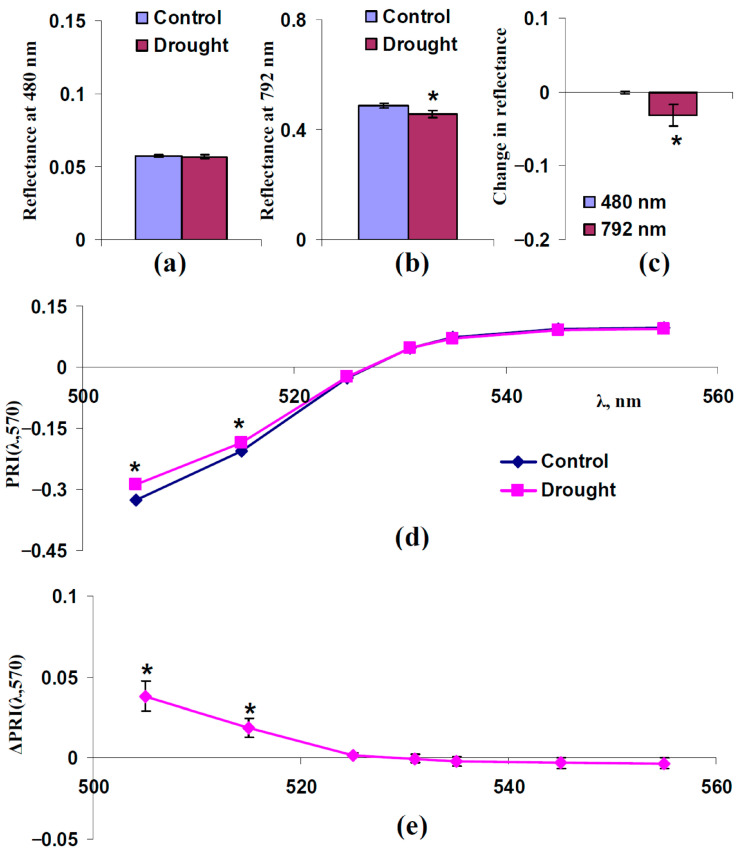
Experimental influence of the 7-day drought on reflectance at 480 and 792 nm and PRI(λ,570) in pea leaves (*n* = 42). Drought was induced by absence of irrigation (the “Drought” group); control plants were irrigated (the “Control” group). (**a**) Reflectance at 480 nm in these groups. (**b**) Reflectance at 792 nm in these groups. (**c**) Changes in reflectance at 480 and 792 nm, which were calculated as the difference between ones in “Drought” and “Control” groups. (**d**) Dependences of PRI(λ,570) on λ in “Drought” and “Control” groups. (**e**) Dependence of changes in PRI(λ,570) (ΔPRI(λ,570)) on λ. ΔPRI(λ,570) were calculated as the difference between PRI(λ,570) in the “Drought” group and PRI(λ,570) in the “Control” group. *, differences between groups are significant (*p* < 0.05).

**Figure 8:**
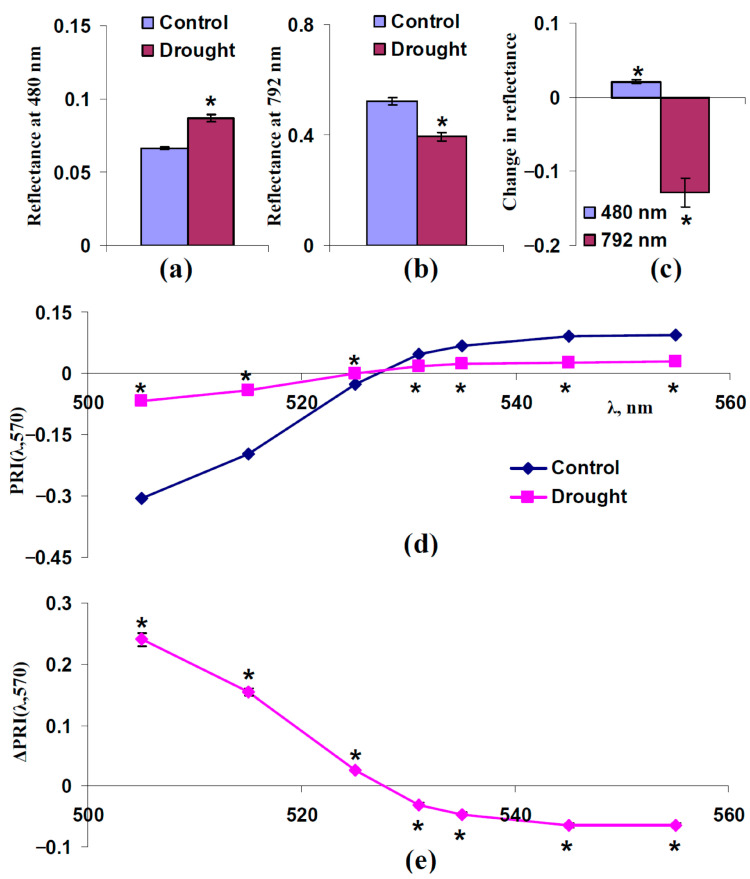
Experimental influence of the 14-day drought on reflectance at 480 and 792 nm and PRI(λ,570) in pea leaves (*n* = 42). Drought was induced by absence of irrigation (the “Drought” group); control plants were irrigated (the “Control” group). (**a**) Reflectance at 480 nm in these groups. (**b**) Reflectance at 792 nm in these groups. (**c**) Changes in reflectance at 480 and 792 nm, which were calculated as the difference between ones in “Drought” and “Control” groups. (**d**) Dependences of PRI(λ,570) on λ in “Drought” and “Control” groups. (**e**) Dependence of changes in PRI(λ,570) (ΔPRI(λ,570)) on λ. ΔPRI(λ,570) were calculated as the difference between PRI(λ,570) in the “Drought” group and PRI(λ,570) in the “Control” group. *, differences between groups are significant (*p* < 0.05).

We hypothesized that changes in the light scattering of the leaf surface and spongy mesophyll layer could participate in changes in typical and modified PRIs under drought. An analysis of two questions was necessary to verify this hypothesis: (i) Could drought increase the reflectance at 480 nm (increase F_S_) and decrease the reflectance at 792 nm (decrease s_Sp_)? (ii) Were increasing PRI(λ,570) at λ < 531 nm and decreasing PRI(λ,570) at λ ≥ 531 nm formed under drought? Figure 7 and Figure 8 show the results of the experimental analysis for moderate (7 days) and strong (14 days) drought, respectively.

It was shown that the 7-day drought significantly decreased the reflectance at 792 nm (Figure 7b,c). This decrease was accompanied by a significant increasing PRI(λ,570) at λ < 531 nm and insignificant decreasing PRI(λ,570) at λ ≥ 531 nm. Considering absence of changes in the reflectance at 480 nm in this case, the results showed that moderate drought was probable to influence typical and modified PRIs (Figure 7d,e) by decreasing s_Sp_ only (changes in F_S_ did not participate in changes in PRIs).

The 14-day drought increased the reflectance at 480 nm and decreased the reflectance at 792 nm; both changes were significant and had large magnitudes (their relative magnitudes were about 25–30%) (Figure 8a–c). The strong drought also induced an increasing PRI(λ,570) at λ < 531 nm and decreasing PRI(λ,570) at λ ≥ 531 nm (Figure 8d,e); the changes were significant and large.

These results supported the participation of increasing F_S_ and decreasing s_Sp_ in changes in typical and modified PRIs under drought. However, the magnitudes of changes in these PRIs under the 14-day drought were very large (Figure 8e) in comparison with the magnitudes of changes induced by increasing F_S_ and decreasing s_Sp_ (Figure 3b and Figure 5b for model-based results; Figure 4e and Figure 6e for experimental results). This point could be explained by the participation of changes in the concentrations of photosynthetic pigments in forming changes in typical and modified PRIs.

### 3.4. Analysis of Participation of Changes in Concentrations of Photosynthetic Pigments in Changes in Typical and Modified Photochemical Reflectance Indices Under Drought

The model-based analysis of the influence of changes in the concentrations of chlorophyll a (Figure S2), chlorophyll b (Figure S3), and carotenoids (Figure S4) showed an absence of typical responses on the long-term stressor action, which was observed in [41,43]: simultaneous increasing PRI(λ,570) at λ < 531 nm and decreasing PRI(λ,570) at λ ≥ 531 nm were not formed. The changes in all investigated PRIs had the same direction for changes in the concentrations of chlorophyll a and b and carotenoids. Thus, the changes in the concentrations of these photosynthetic pigments probably did not participate in the influence of drought (and, maybe, other long-term stressors) for typical and modified PRIs.

**Figure 9:**
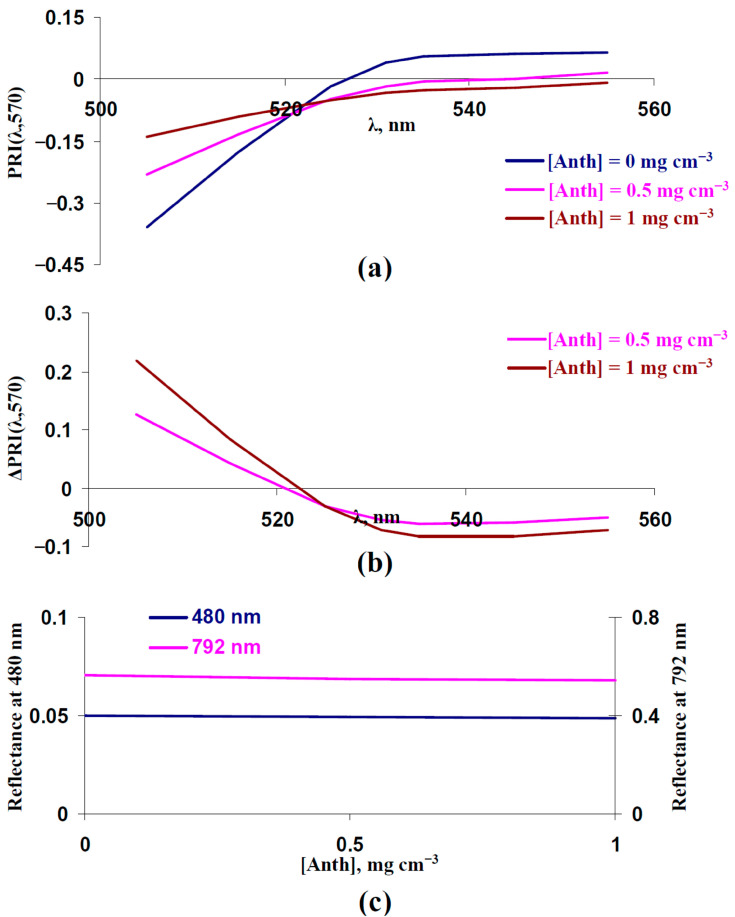
Influence of average concentration of anthocyanin ([Anth]) on PRI(λ,570) and reflectance at 480 and 792 nm. Results of model-based calculation are shown. (**a**) Model-based dependences of PRI(λ,570) on λ, which were calculated at [Anth] = 0 mg cm^−3^ (basic value), [Anth] = 0.5 mg cm^−3^ (high value 1), and [Anth] = 1 mg cm^−3^ (high value 2). Other parameters of the model of light reflectance and transmittance in plant leaves were basic (Table 1). (**b**) Dependences of changes in PRI(λ,570) (ΔPRI(λ,570)) on λ. ΔPRI(λ,570) were calculated as the difference between PRI(λ,570) at [Anth] = 0.5 mg cm^−3^ or [Anth] = 1 mg cm^−3^ and PRI(λ,570) at [Anth] = 0 mg cm^−3^. (**c**) Dependences of reflectance at 480 and 792 nm on [Anth].

In contrast, increasing the concentration of anthocyanin strongly influenced typical and modified PRIs (Figure 9a,b): an increasing PRI(λ,570) at λ < 525 nm and decreasing PRI(λ,570) at λ ≥ 525 nm were formed. The large magnitudes of the changes (Figure 9b) were similar to the experimental changes after the 14-day drought (Figure 8c). Changes in the reflectance at 480 and 792 nm were absent (Figure 9c).

Thus, we supposed that increasing the anthocyanin concentration could be the additional mechanism of changes in typical and modified PRIs under the action of the long-term stressors (drought). An experimental analysis of the changes in anthocyanin concentration under drought could support this proposition. We analyzed anthocyanin reflectance indices 1 (ARI1) and 2 (ARI2) after the 7-day and 14-day drought to verify this point. In accordance with [77,78], these indices are strongly sensitive to anthocyanin concentration.

**Figure 10:**
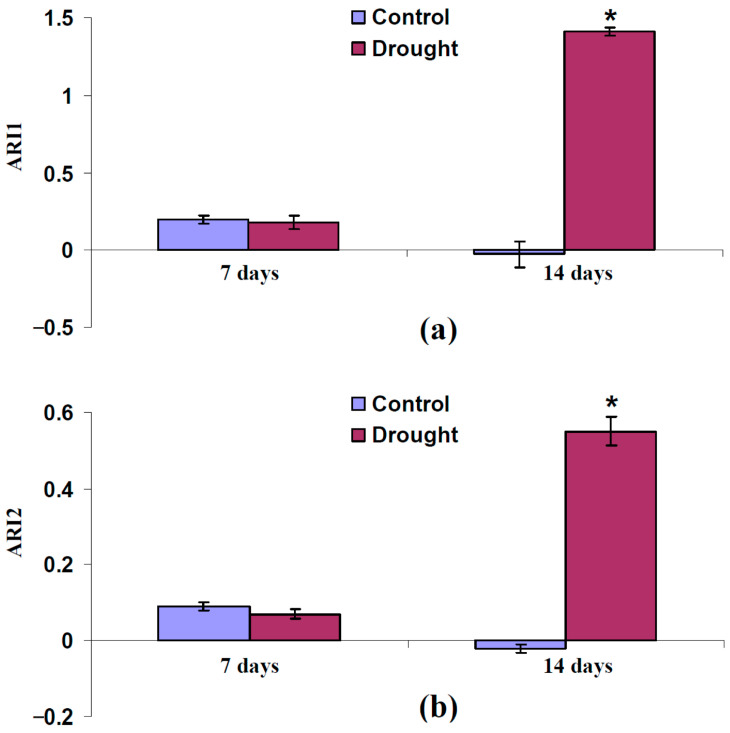
Experimental influence of 7-day and 14-day drought on anthocyanin reflectance index 1 (ARI1) (**a**) and 2 (ARI2) (**b**) in pea leaves (*n* = 42). Drought was induced by absence of irrigation (the “Drought” group); control plants were irrigated (the “Control” group). ARI1 and ARI2 were measured using handheld PolyPen RP 410 UVIS system. *, differences between groups are significant (*p* < 0.05).

It was shown (Figure 10) that ARI1 and ARI2 were not significantly changed after the 7-day drought. In contrast, the 14-day drought strongly increased both ARI1 and ARI2, which showed an increasing anthocyanin concentration in the leaves of pea plants. The last result supported the participation of increasing anthocyanin concentrations in changes in typical and modified PRIs under the action of a strong drought.

### 3.5. Analysis Changes in Typical and Modified Photochemical Reflectance Indices Under Increased Concentrations of Carotenoids

**Figure 11:**
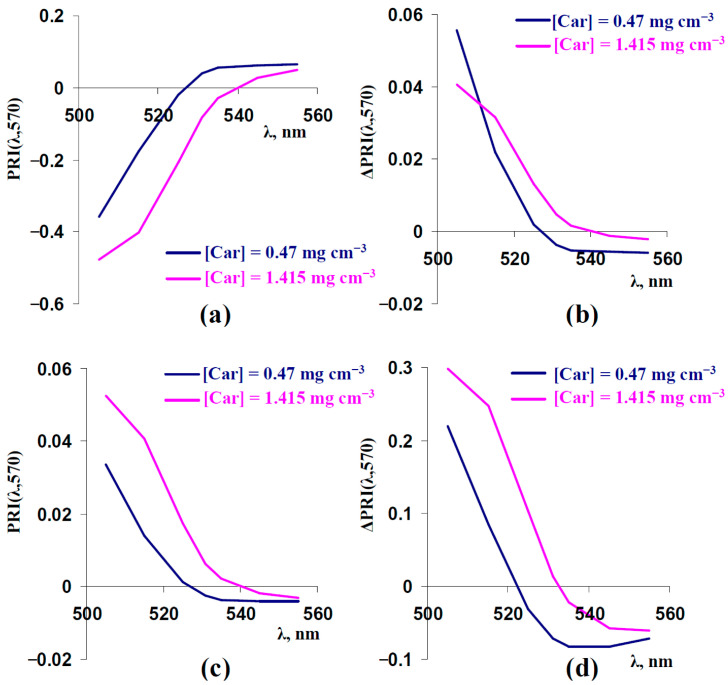
(**a**) Model-based dependences of PRI(λ,570) on λ, which were calculated at [Car] = 0.47 mg cm^−3^ (basic value) and [Car] = 1.415 mg cm^−3^. (**b**) Model-based dependences of F_S_-induced changes in PRI(λ,570) (ΔPRI(λ,570)) on λ at [Car] = 0.47 mg cm^−3^ (basic value) and [Car] = 1.415 mg cm^−3^. ΔPRI(λ,570) were calculated as the difference between PRI(λ,570) at F_S_ = 0.225 and PRI(λ,570) at F_S_ = 0.15. (**c**) Model-based dependences of s_Sp_-induced ΔPRI(λ,570) on λ at [Car] = 0.47 mg cm^−3^ (basic value) and [Car] = 1.415 mg cm^−3^. ΔPRI(λ,570) were calculated as the difference between PRI(λ,570) at s_Sp_ = 300 cm^−1^ and PRI(λ,570) at s_Sp_ = 600 cm^−1^. (**d**) Model-based dependences of [Anth]-induced ΔPRI(λ,570) on λ at [Car] = 0.47 mg cm^−3^ (basic value) and [Car] = 1.415 mg cm^−3^. ΔPRI(λ,570) were calculated as the difference between PRI(λ,570) at [Anth] = 1 mg cm^−3^ and PRI(λ,570) at [Anth] = 0 mg cm^−3^. Other parameters of the model of light reflectance and transmittance in plant leaves were basic (Table 1).

Results of the current investigation showed that PRIs with negative initial values were mainly increased under increasing F_S_ and [Anth] and under decreasing s_Sp_; in contrast, PRIs with positive initial values were mainly decreased in these cases (Figure 3, Figure 5, and Figure 9). As a result, we supposed that the sign of the initial value of the typical or modified photochemical reflectance index influenced the direction of changes in this index. In accordance with Figure S4a, PRIs with negative values were transformed into PRIs with positive values at increasing concentrations of carotenoids. Considering this point, we additionally analyzed the influence of F_S_, s_Sp_, and [Anth] on typical and modified PRIs at a strongly increased concentration of carotenoids ([Car] = 1.415 mg cm^−3^) (Figure 11).

It was shown that increasing the concentration of carotenoids transformed PRI(531,570) and PRI(535,570) from positive to negative (Figure 11a). The model-based dependences of changes in PRI(λ,570) (ΔPRI(λ,570)) on λ, which were induced by increasing F_S_ (Figure 11b), decreasing s_SP_ (Figure 11c), and increasing [Anth], were in good accordance with the sign of the PRI: the increase in [Car] and transformation of the PRI from positive to negative mainly caused an inverting of the direction of the index changes.

## 4. Discussion

Remote sensing plays an important role in timely protecting plants under the action of stressors. Narrowband and broadband reflectance indices [15,16], which are mainly calculated on the basis of the reflectance at two or three spectral bands, are widely used to estimate specific plant characteristics. The PRI, which is calculated on the basis of the reflectance at 531 and 570 nm [31,32,33], is considered to be one of the key indices for plant remote sensing [34,35,36].

Potentially, PRI [31,32,33,34,35,36] and its modifications with short or long measuring wavelengths [41,43,64] can be an effective tool for estimating photosynthetic changes. Experimental investigations show that the fast light-induced decreasing of typical [32,37,55] and modified [64] PRIs is strongly related to quickly decreasing photosynthetic activity. As a result, the parameters of this activity can be potentially calculated as linear functions of PRIs under the short-term action of the excess light [64]. In contrast, the relationships between PRIs and parameters of photosynthesis can be intricated on large time intervals (days or more); e.g., PRIs with short measuring wavelengths (<about 531 nm) and with long measuring wavelengths (>about 531 nm) are increased and decreased, respectively, under the long-term action of drought [41] or salinization [43], providing opposite directions of these relationships for different modified PRIs. The typical PRI can be both increased [41,55] and decreased [41] under drought in different experiments.

Different responses of PRIs on the short-term and long-term action of stressors are likely to be related to the different mechanisms of changes in these indices. The fast PRI decrease is caused by decreasing pH in the chloroplast lumen, which induces transitions in the xanthophyll cycle [31,32,33,47] and chloroplast shrinkage [35,37]. These mechanisms were shown for the typical PRI [35,36]; however, modified PRIs with short and long measuring wavelengths are likely to be dependent on similar processes [64]. The slow changes in the typical PRI are considered to be caused by long-term changes in the concentrations of chlorophylls and carotenoids [56,57,58,59]; the participation of changes in light scattering in leaves cannot be also excluded [58]. Potentially, slow changes in modified PRIs under long-term stressor action can have the same mechanisms [41,43]; however, this problem is weakly investigated. Changes in the concentration of anthocyanin, which influences the reflectance at both 531 and 570 nm and modifies the relationships of PRIs to the concentrations of chlorophylls and carotenoids [59], can be another mechanism of slow changes in typical and modified PRIs.

Thus, our work analyzes the influence of the light scattering of leaf surfaces and mesophyll layers and concentrations of photosynthetic pigments on typical and modified PRIs. The analysis is based on the previously developed analytical model of light reflectance and transmittance in the leaves of dicot plants [67]. Experimental results are used to verify model-based results and to additionally analyze the mechanisms of the long-term action of stressors (drought) on typical and modified PRIs.

First, we show that both increasing light scattering of the leaf surface (increasing F_S_) (Figure 3 and Figure 4) and decreasing this scattering in the spongy mesophyll (decreasing s_Sp_) (Figure 5 and Figure 6) increases PRI(λ,570) at λ < 531 nm and decreases PRI(λ,570) at λ ≥ 531 nm. These changes in PRIs are in good accordance with the experimental results in pea leaves under the action of drought [41], heating [41], and salinization [43]. The current experimental analysis additionally shows that a moderate drought induces a weak decrease in the light scattering coefficient (Figure 7c) and a strong drought induces a large decrease in this coefficient and increase in the fraction of the rough leaf surface (Figure 8c). An increasing PRI(λ,570) at λ < 531 nm and decreasing PRI(λ,570) at λ ≥ 531 nm are also observed under a moderate drought (with low magnitudes of changes) (Figure 7e) and under a strong drought (with high magnitudes of changes) (Figure 8e). The results support the hypothesis about the influence of light scattering on the PRI [58]; however, they show that the influences of changes in the light scattering of the leaf surface and light scattering of the spongy mesophyll are opposite.

It should be noted that the current investigation does not explain the mechanisms of influence of drought on light scattering. The drought-induced increase in light scattering on the leaf surface, which is caused by an increasing fraction of the rough surface, seems to be expected because water loss and turgor decreasing contribute to leaf wrinkling. In contrast, the drought-induced decrease of light scattering in the spongy mesophyll requires future experimental and model-based investigations. It can be speculated that this effect is caused by drought-induced changes in the air space in leaves, cell size, and/or vacuole/cytoplasm ratio; however, characteristics of these changes and their relationships to light scattering are not clear.

Second, changes in the concentrations of chlorophylls a and b and carotenoids do not induce changes in typical and modified PRIs, which are mainly observed in experiments [41,43]. The decreasing concentrations of chlorophylls a (Figure S2) and b (Figure S3) decrease typical and modified PRIs and vice versa. The decreasing concentration of carotenoids (Figure S4) increases typical and modified PRIs and vice versa. These effects can be explained by different light absorptions (see [66,67] or Figure 1b) in spectral bands, which are used to calculate typical and modified PRIs [43]: chlorophylls have a relatively high absorption at 570 nm (i.e., decreasing their concentrations should increase R_570_ and, therefore, decrease PRIs); in contrast, the light absorption of carotenoids is minimal at 570 nm (i.e., increasing their concentration should decrease reflectance at measuring wavelengths and, therefore, decrease PRIs). Thus, the results show that changes in the concentrations of these pigments, which are considered as important mechanisms of slow changes in the typical PRI [56,57,58,59], are not likely to be the main mechanism of changes in modified PRIs.

In contrast, increasing the concentration of anthocyanin, which can absorb light in the broad green–yellow spectral region [59,66,77], strongly increases PRI(λ,570) at λ < 525 nm and decreases PRI(λ,570) at λ ≥ 525 nm (Figure 9b). The results are similar to experimental changes in typical and modified PRIs under the long-term action of stressors ([41,43] and Figure 8e). Moreover, our experimental results (Figure 10) show that the 14-day drought induces a large increase in ARI1 and ARI2, which are strongly related to the anthocyanin concentration [77,78]. The last result is in good accordance with increasing this concentration under drought, which was earlier shown [74,75], and supports the participation of anthocyanin in drought-induced changes in typical and modified PRIs.

Third, an analysis of the model shows that the direction of changes in typical and modified PRIs is dependent on the initial sign of their indices (Figure 11). Particularly, increasing the light scattering of the leaf surface and the anthocyanin concentration and decreasing the light scattering of the spongy mesophyll decreases the typical PRI at [Car] = 0.47 mg cm^−3^ (Figure 3b, Figure 5b, and Figure 9b) and increases this index at [Car] = 1.415 mg cm^−3^ (Figure 11b–d). Potentially, the effect can be a mechanism of different directions of changes in the typical PRI under the long-term action of stressors (e.g., drought can both increase [55] and decrease [79] the typical PRI in pea leaves in different experiments). Additionally, the results show that the combination of changes in the concentrations of carotenoids (and, maybe, chlorophylls a and b) and changes in light scattering and anthocyanin concentration can intricately influence the typical PRI. The last point is in good accordance with disruptions of the relationships of the concentrations of carotenoids and chlorophylls a and b with the typical PRI at an increased concentration of anthocyanin in leaves [59].

**Figure 12:**
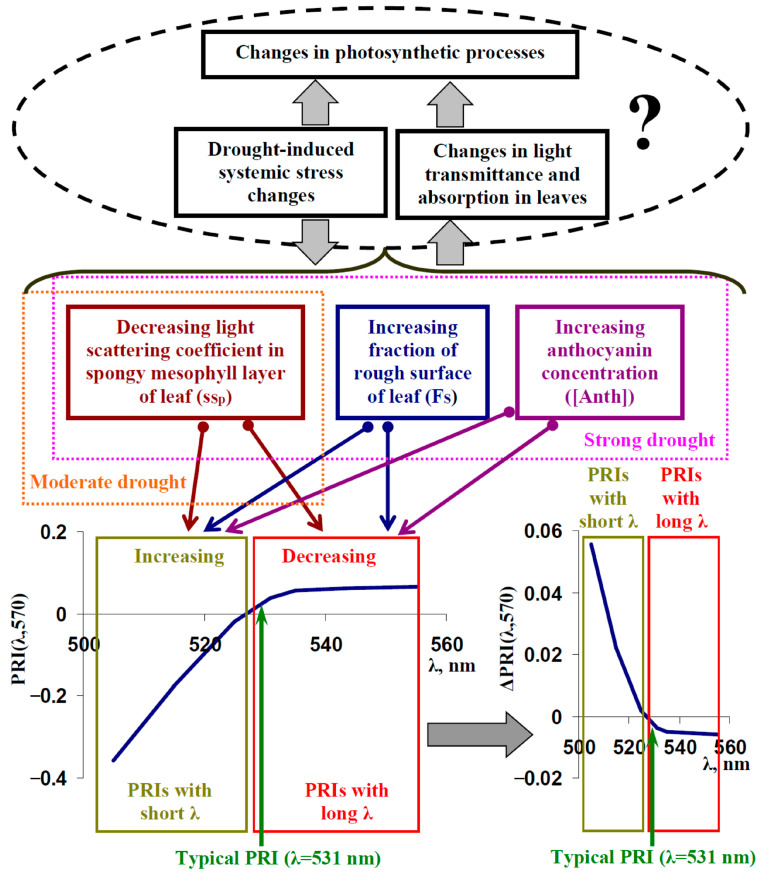
Scheme of influence of drought on typical and modified photochemical reflectance indices (PRIs) with short and long measuring wavelengths (λ) in leaves of dicot plants through changes in the fraction of the rough surface, light scattering coefficient in the spongy mesophyll layer, and the anthocyanin concentration. A region surrounded by a dotted line shows possible relationships of the long-term changes in typical and modified PRIs with photosynthetic parameters. Strong drought increases the fraction of the rough surface and the anthocyanin concentration and decreases the light scattering coefficient in the spongy mesophyll layer. Moderate drought is probable to only decrease the light scattering coefficient in the spongy mesophyll layer. ΔPRI(λ,570) shows changes in typical and modified PRIs under action of drought.

Thus, the combination of the model-based and experimental analysis shows the relationships of light scattering and anthocyanin concentration with typical and modified PRIs, which can participate in the influence of long-term stressors (e.g., drought) on these PRIs (Figure 12). It is important that the results of the model-based and experimental analyses are in good accordance without a specific description of the mechanisms of fast changes in PRI [31,32,33,37,47] in the developed model of light reflectance and transmittance in the leaves of dicot plants (File S1, [67]). The last point means that changes in the reflectance in the broad green–yellow spectral region (light scattering of the leaf surface and spongy mesophyll influences all reflectance spectrum [67]; anthocyanin influences reflectance at 500–600 nm [59,77]) can be effective mechanisms for the responses of typical and, especially, modified PRIs under the long-term action of stressors.

Our previous results show that changes in typical and modified PRIs under the action of drought [41], salinization [43], and heating [41] are strongly related to photosynthetic parameters. However, the current results show that changes in light scattering and the anthocyanin concentration, which influence reflectance in the broad spectral range [59,66,67], seem to be an important mechanism of changes in these PRIs. This means that the relationships of typical and modified PRIs with photosynthetic parameters cannot be based on changes in specific narrow spectral bands, which are mainly considered as the main reason of photosynthetic influence on the typical PRI [30,31,33,64]. As a result, the potential mechanisms of the relationships of typical and modified PRIs with photosynthetic parameters should be discussed in the current work.

Two possible ways of forming relationships between long-term changes in typical and modified PRIs and photosynthetic parameters under the long-term action of stressors (particularly drought, as investigated in the current work) can be speculated (Figure 12). (i) Drought-induced systemic stress changes can simultaneously modify light scattering and the concentrations of anthocyanin and suppress photosynthetic processes (e.g., through damage of photosynthetic machinery, which can be caused by drought [41,79]). A simultaneous forming of these changes, which are caused by the same reason, can provide relationships between the investigated values. (ii) Changes in light scattering (increasing F_S_ and decreasing s_Sp_) and the concentration of anthocyanin should influence light transmittance and absorption in leaves [59,67]. Considering the key role of light for photosynthesis [80,81,82], changes in transmittance and absorption can strongly influence photosynthetic processes. This mechanism can also influence the relationships of typical and modified PRIs with photosynthetic parameters.

Finally, it should be noted that modifying light scattering and anthocyanin concentration can be a factor influencing the measurements of short-term light-induced changes in PRIs. These changes, which can be calculated as the differences between PRIs under light and dark conditions [51,83,84], are widely used to estimate photosynthetic parameters based on typical PRIs [47,52,55,60,61,62], since they are considered to be independent of slow changes in the photochemical reflectance index. A direct estimation of the “dark” PRI is a difficult problem in the remote sensing of plants in fields; as a result, the PRI can be calculated on the basis of other vegetation reflectance indices [51,83]. Our results show that an estimation of the dark PRI can be modified by light scattering and the anthocyanin concentration; i.e., these factors should also be considered. Moreover, it cannot be fully excluded that the initial light scattering and anthocyanin concentration can influence the parameters of light-induced changes in the PRI (e.g., magnitude of the changes). Including the fast changes in typical and modified PRIs into the model of light reflectance and transmittance in the leaves of dicot plants can provide a theoretical tool to check this hypothesis in the future.

As a whole, our work shows that broadband shifts in reflectance at 500–600 nm, which are caused by changes in the light scattering of the leaf surface and spongy mesophyll and changes in the anthocyanin concentration, can be mechanisms of slow changes in typical and modified PRIs caused by the long-term action of stressors (at least, drought). The effects of these shifts do not require fast changes in light absorption, induced by the photosynthetic decreasing of pH in the chloroplast lumen and the following transitions in the xanthophyll cycle [31,32,33,47] and chloroplast shrinkage [35,37]. However, our previous results [41,43] show that these slow changes in typical and modified PRIs are related to photosynthetic parameters. Experimental and model-based analyses of the mechanisms of these relationships are an important task for future investigations.

## 5. Materials and Methods

### 5.1. Plant Cultivation and Drought

Peas (*Pisum sativum* L., cultivar “Falyonsky Yubileyniy”) were used in experiments because our analytical model of light reflectance and transmittance was earlier parameterized and verified on the basis of pea leaves, which had both palisade and spongy mesophyll layers [67]. As a result, using pea leaves was optimal for comparing experimental and model-based results.

Plants were cultivated in pots, which contained the peat soil “Morris Green” (Pelgorskoe M, Ryabovo, Russia); nine plants were cultivated in each pot. Plants were illuminated by luminescent lamps FSL YZ18RR (Foshan Electrical And Lighting Co., Ltd., Foshan, China). Irrigation was performed three times a week.

Experiments were initiated after 14 days of plant cultivation. Termination of irrigation was used to induce drought; control plants were irrigated. The maximal drought duration was 14 days because the 14-day drought markedly reduced turgor of pea leaves; as a result, reflectance measurements in leaves were difficult after more than 14 days of drought. Final relative water contents in shoots (RWC) were calculated on the basis of their dry (DW) and fresh (FW) weights in accordance with Equation (3):

(3) $$\mathrm{RWC}=100\frac{\mathrm{FW}-\mathrm{DW}}{\mathrm{FW}}$$

The dry weights were measured after heating for 2 h at 100 °C in the TV-20-PZ-K thermostat (Kasimov Instrument Plant, Kasimov, Russia). Final water contents were 67.4 ± 2.2% in shoots of plants under drought and 90.4 ± 0.9% in the shoots of plants under irrigation.

### 5.2. Measurements of Reflectance of Leaves and Calculation of Reflectance Indices

Reflectance spectra were measured in the central part of second pea leaves; the leaves were intact and were not cut off from plants. The handheld PolyPen RP 410 UVIS system (Photon Systems Instruments, Drásov, Czech) was used to these measurements.

Equation (2) was used to calculate typical and modified photochemical reflectance indices. Measuring wavelengths were 531 nm (for the typical PRI) and 505, 515, 525, 535, 545, and 555 nm (for modified PRIs). In accordance with [41,43], modified PRIs calculated on the basis of these measuring wavelengths are sensitive to long-term action of drought and salinization.

Our model-based analysis showed [67] that the reflectance at 480 nm and that at 792 nm are mainly related to light scattering of the leaf surface, which is based on F_S_, and to light scattering of the spongy mesophyll layer, which is based on s_Sp_, respectively. As a result, reflectance at 480 nm and reflectance at 792 nm were used in the analysis to estimate these characteristics.

To experimentally estimate the influence of light scattering on typical and modified PRIs in leaves of control pea plants (under irrigation), individual experimental results were divided into two equal groups: “Low”, which includes plants with reflectance at 480 nm (for light scattering of the leaf surface) or at 792 nm (for light scattering in the spongy mesophyll) lower than the median of this reflectance, and “High”, which includes plants with reflectance at 480 nm or at 792 nm higher than the median of this reflectance. The medians for reflectance at 480 nm or at 792 nm were calculated on the basis of all plants under irrigation. After that, investigated reflectance parameters (typical and modified PRIs, reflectance at 480 and 792 nm) were compared in these groups.

To experimentally estimate the influence of light scattering on typical and modified PRIs in peas under drought, these PRIs and the reflectance at 480 and 792 nm were compared in experimental and control plants after the 7-day and 14-day drought. Earlier, we showed that the 7-day drought weakly influenced photosynthetic machinery in pea plants; in contrast, the 14-day drought strongly damaged this machinery [41].

Additionally, anthocyanin reflectance indices 1 (ARI1) and 2 (ARI2), which show the concentration of anthocyanin in leaves [77,78], were analyzed in the current work. These indices were automatically calculated by software of the PolyPen RP 410 UVIS system in accordance with equations developed in [77,78]:

(4) $$\mathrm{ARI}1=\frac{1}{R_{550}}-\frac{1}{R_{700}}$$

(5) $$\mathrm{ARI}2=R_{790}\left(\frac{1}{R_{550}}-\frac{1}{R_{700}}\right)$$

where R_790_, R_700_, and R_550_ are the reflectance at 790, 700, and 550 nm.

## 6. Conclusions

Long-term changes in typical and modified photochemical reflectance indices are an important factor that modifies efficiency of using PRIs in plant remote sensing. The results of our current work showed that increasing the light scattering of the leaf surface and the anthocyanin concentration and decreasing the light scattering of the spongy mesophyll increased PRIs with short measuring wavelengths and decreased PRIs with long measuring wavelengths. The action of drought induced similar changes in typical and modified PRIs, which were also accompanied by an increase in the light scattering of the leaf surface and the anthocyanin concentration and a decrease in the light scattering of the spongy mesophyll.

These results show that processes which modify reflectance in the broad spectral range (500–600 nm) can be mechanisms of slow changes in typical and modified PRIs, including drought-induced changes. These mechanisms do not require the photosynthetic acidification of the chloroplast lumen; however, our previous results showed that the slow changes in typical and modified PRIs can be related to the parameters of photosynthesis. The mechanisms which are the basis of these relationships require future experimental and model-based investigations.

## Figures and Tables

**Table 1:** Basic parameters that were used in the analytical model of light reflectance and transmittance in leaves of dicot plants. Parameters are described in File S1, “Equations of the analytical model of light reflectance and transmittance in leaf of dicot plants”, in detail. Values of parameters from our previous work [67] were mainly used; basic concentration of anthocyanin was assumed to be equal to zero.

Parameter	Value	Unit
Intensity of the forward-collimated light directed to adaxial leaf surface (*I*_0_)	1000	µmol m^−2^s^−1^
Angle of light incidence on adaxial leaf surface (*β_O_*_1_)	35	°
Intensity of the backward-collimated light directed to abaxial leaf surface (*J*_0_)	0	µmol m^−2^s^−1^
Angle of light incidence on abaxial leaf surface (*β_O_*_2_)	35	°
Quantity of iterations which is necessary to approximately describe the reflectance and transmittance spectra of leaves (N)	6	-
Refractive index in air (*n_O_*)	1	-
Refractive index in leaf (*n_I_*)	1.415	-
Transmittance coefficient for the scattered light transfer from air to leaf (*T_s_^OI^*)	0.866	-
Transmittance coefficient for the scattered light transfer from leaf to air (*T_s_^IO^*)	0.469	-
Fraction of the rough surface (*F_S_*)	0.15	-
Thickness of the palisade mesophyll layer (*h*)	35.5	µm
Thickness of the spongy mesophyll layer (*l*)	58.6	µm
Light scattering coefficient in the palisade mesophyll layer ($s_{P}$)	5	cm^−1^
Light scattering coefficient in the spongy mesophyll layer ($s_{Sp}$)	600	cm^−1^
Asymmetry factor (*f*)	0.5	-
Average concentration of chlorophyll a ([Chl a])	1.6	mg cm^−3^
Average concentration of chlorophyll b ([Chl b])	1.05	mg cm^−3^
Average concentration of carotenoids ([Car])	0.47	mg cm^−3^
Average concentration of anthocyanin ([Anth])	0	mg cm^−3^
Ratio between concentrations of photosynthetic pigments in the spongy and palisade mesophyll (*N_Sp_*_/*P*_)	0.2	-

## Data Availability

The original contributions presented in the study are included in the article/Supplementary Material, further inquiries can be directed to the corresponding author/s.

## Supplementary Materials

The following supporting information can be downloaded at https://www.mdpi.com/article/10.3390/plants14213255/s1, File S1: Equations of the analytical model of light reflectance and transmittance in leaves of dicot plants; Figure S1: Influence of light scattering coefficient in the palisade mesophyll layer (s_P_) on PRI(λ,570); Figure S2: Influence of average concentration of chlorophyll a ([Chl a]) on PRI(λ,570); Figure S3: Influence of average concentration of chlorophyll b ([Chl b]) on PRI(λ,570); Figure S4: Influence of average concentration of carotenoids ([Car]) on PRI(λ,570).

## Abbreviations

The following main abbreviations are used in this manuscript:

PRI	Photochemical reflectance index
R_531_	Light reflectance at 531 nm
R_570_	Light reflectance at 570 nm
PRI(λ,570)	Photochemical reflectance index with measuring wavelength equaling to λ
ΔPRI(λ,570)	Changes in PRI(λ,570)
ARI1	Anthocyanin reflectance index 1
ARI2	Anthocyanin reflectance index 2
F_S_	Fraction of the rough surface
s_Sp_	Light scattering coefficient in the spongy mesophyll layer
[Anth]	Average concentration of anthocyanin
[Chl a]	Average concentration of chlorophyll a
[Chl b]	Average concentration of chlorophyll b
[Car]	Average concentration of carotenoids
