# Supplementary material for: Light Scattering of Leaf Surface and Spongy Mesophyll and Concentration of Anthocyanin Influence Typical and Modified Photochemical Reflectance Indices
Source: Plants (Basel). 2025 Oct 24;14(21):3255. doi: 10.3390/plants14213255 (PMC12609760; doi:10.3390/plants14213255)
Supplement: Supplementary file 1 [file plants-14-03255-s001.zip › Figure S1.pdf]

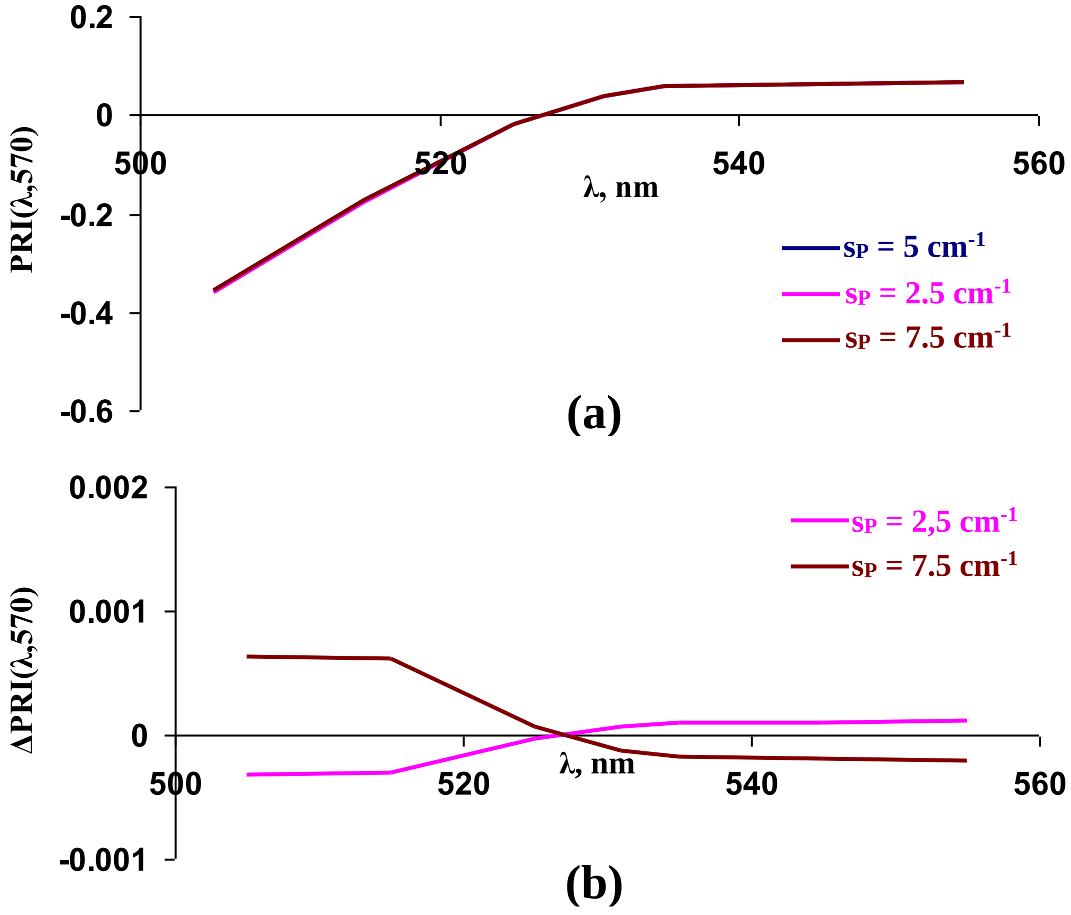

Figure S1. Influence of light scattering coefficient in the palisade mesophyll layer (s_P) on PRI(λ,570). Results of model-based calculation are shown, **(a)** Model-based dependences of PRI(λ,570) on λ, which were calculated at s_P = 5 cm^{-1} (basic value), s_P = 2.5 cm^{-1} (low value), and s_P = 7.5 cm^{-1} (high value). Other parameters of the model of light reflectance and transmittance in plant leaf were basic (Table 1). **(b)** Dependences of changes in PRI(λ,570) (ΔPRI(λ,570)) on λ. ΔPRI(λ,570) were calculated as difference between PRI(λ,570) at s_P = 2.5 cm^{-1} or s_P = 7.5 cm^{-1} and PRI(λ,570) at s_P = 5 cm^{-1}.
