# Supplementary material for: Light Scattering of Leaf Surface and Spongy Mesophyll and Concentration of Anthocyanin Influence Typical and Modified Photochemical Reflectance Indices
Source: Plants (Basel). 2025 Oct 24;14(21):3255. doi: 10.3390/plants14213255 (PMC12609760; doi:10.3390/plants14213255)
Supplement: Supplementary file 1 [file plants-14-03255-s001.zip › Figure S4.pdf]

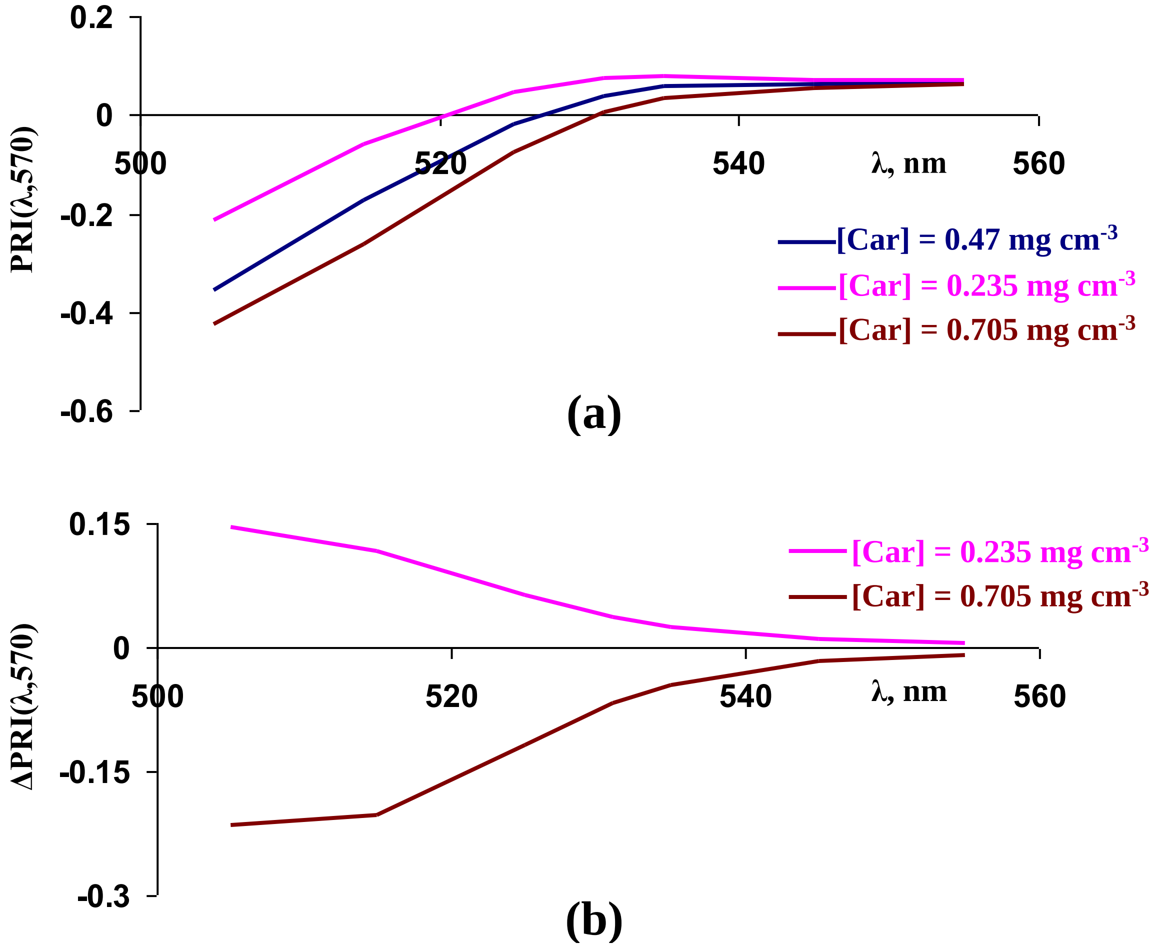

Figure S4. Influence of average concentration of carotenoids ([Car]) on PRI(λ,570). Results of model-based calculation are shown, **(a)** Model-based dependences of PRI(λ,570) on λ, which were calculated at [Car] = 0.47 mg cm^{-3} (basic value), [Car] = 0.235 mg cm^{-3} (low value), and [Car] = 0.705 mg cm^{-3} (high value). Other parameters of the model of light reflectance and transmittance in plant leaf were basic (Table 1). **(b)** Dependences of changes in PRI(λ,570) (ΔPRI(λ,570)) on λ. ΔPRI(λ,570) were calculated as difference between PRI(λ,570) at [Car] = 0.235 mg cm^{-3} or [Car] = 0.705 mg cm^{-3} and PRI(λ,570) at [Car] = 0.47 mg cm^{-3}.
