# Supplementary material for: Light Scattering of Leaf Surface and Spongy Mesophyll and Concentration of Anthocyanin Influence Typical and Modified Photochemical Reflectance Indices
Source: Plants (Basel). 2025 Oct 24;14(21):3255. doi: 10.3390/plants14213255 (PMC12609760; doi:10.3390/plants14213255)
Supplement: Supplementary file 1 [file plants-14-03255-s001.zip › File S1.pdf]

Supplementary Materials

File S1. Equations of the analytical model of light reflectance and transmittance in leaf of dicot plants

The analytical model of light reflectance and transmittance in leaf of dicot plants was described in detail in our previous work (Sukhova et al., Plants, 2024, 13, 3258, doi: 10.3390/plants13223258). Figure 1a shows the scheme of the model. Equations of the model are shown below; parameters of the models are shown in Table S1.

Basic variables of the model

The leaf was assumed as an optical system including two main optical layers: the palisade mesophyll layer with high light absorption (a_P) and low light scattering (s_P) coefficients and the spongy mesophyll layer with high light absorption (a_{Sp}) and high light scattering (s_{Sp}) coefficients. Epidermal layers were not considered in the model. Borders "air-leaf" and "leaf-air" were additionally described in the model. Four variables were considered in the current work (Figure 1a) including intensities of the forward collimated light (I_C), forward scattered light (I_S), backward collimated light (J_C), and backward scattered light (J_S).

Equations describing light reflectance and transmittance on borders "air-leaf" and "leaf-air"

It was assumed that there were two types of transmittances of the collimated light across borders "air-leaf" and "leaf-air": (i) across a smooth surface (without scattering) and (ii) across a rough surface (with scattering). The fraction of the rough surface (F_S) was the model parameter; the fraction of the smooth surface was calculated as $1\text{-}F_S$.

Relationships between the angles of incidence and refraction were described by equations (S1) and (S2), which were based on Snell's law:

$$\beta_{I1} = \arcsin\left(\frac{n_O}{n_I}\sin(\beta_{O1})\right) \quad \text{(S1)}$$

$$\beta_{I2} = \arcsin\left(\frac{n_O}{n_I}\sin(\beta_{O2})\right) \quad \text{(S2)}$$

where n_O and n_I are the refractive indices in air (n_O=1) and in leaf (n_I=1.415), β_{O1} and β_{O2} are the angles of light incidence on adaxial and abaxial leaf surfaces, respectively, β_{I1} and β_{I2} are the angles of refraction under adaxial and abaxial leaf surfaces, respectively.

Fresnel's law-based equations (S3) and (S4) were used for calculation of transmittance coefficients for the collimated light transfer from air to leaf across the smooth surfaces:

$$T_{Ic}^{OI} = 1 - \frac{1}{2}\left[\left(\frac{\sin(\beta_{O1}-\beta_{I1})}{\sin(\beta_{O1}+\beta_{I1})}\right)^2 + \left(\frac{\tan(\beta_{O1}-\beta_{I1})}{\tan(\beta_{O1}+\beta_{I1})}\right)^2\right] \quad \text{(S3)}$$

$$T_{Jc}^{OI} = 1 - \frac{1}{2}\left[\left(\frac{\sin(\beta_{O2}-\beta_{I2})}{\sin(\beta_{O2}+\beta_{I2})}\right)^2 + \left(\frac{\tan(\beta_{O2}-\beta_{I2})}{\tan(\beta_{O2}+\beta_{I2})}\right)^2\right] \quad \text{(S4)}$$

where T_{Ic}^{OI} and T_{Jc}^{OI} are transmittance coefficients for the collimated light transfer from air to leaf on adaxial and abaxial surfaces, respectively. Thus, Equations (S5) and (S6) were used for calculation of intensity of the collimated light transmittance into leaf across adaxial and abaxial surfaces ($I_C(0)$ and $J_C(h+l)$, respectively):

$$I_C(0) = I_0 \cdot (1-F_S) \cdot T_{Ic}^{OI} \quad \text{(S5)}$$

$$J_C(h+l) = J_0 \cdot (1-F_S) \cdot T_{Jc}^{OI} \quad \text{(S6)}$$

where I_0 and J_0 are intensities of the forward and backward collimated light directed to adaxial and abaxial leaf surfaces (the incident light), h is the thickness of the palisade mesophyll layer, l is the thickness of the spongy mesophyll layer.

Equations (S7) and (S8) were used for calculation of intensity of the collimated light reflecting from adaxial and abaxial leaf surfaces in air (J_C^{RO} and I_C^{RO}, respectively):

$$J_C^{RO} = I_0 \cdot (1-F_S)\cdot\left(1-T_{Ic}^{OI}\right) \quad \text{(S7)}$$

$$I_C^{RO} = J_0 \cdot (1-F_S)\cdot\left(1-T_{Jc}^{OI}\right) \quad \text{(S8)}$$

Equations (S9) and (S10) were used for calculation of transmittance coefficients for collimated light transfer from leaf to air:

$$T_{Ic}^{IO} = 1-\frac{1}{2}\left[\left(\frac{\sin(\beta_{I1}-\beta_{O1})}{\sin(\beta_{I1}+\beta_{O1})}\right)^2+\left(\frac{\tan(\beta_{I1}-\beta_{O1})}{\tan(\beta_{I1}+\beta_{O1})}\right)^2\right] \quad \text{(S9)}$$

$$T_{Jc}^{IO} = 1-\frac{1}{2}\left[\left(\frac{\sin(\beta_{I2}-\beta_{O2})}{\sin(\beta_{I2}+\beta_{O2})}\right)^2+\left(\frac{\tan(\beta_{I2}-\beta_{O2})}{\tan(\beta_{I2}+\beta_{O2})}\right)^2\right] \quad \text{(S10)}$$

where T_{Ic}^{IO} and T_{Jc}^{IO} are the transmittance coefficients for the collimated light transfer from leaf to air on abaxial and adaxial leaf surfaces, respectively. Equations (S9) and (S10) could not be solved at β_{I1} and β_{I2} which were more than about 45° because light was fully reflected in this case; the case was considered to describe transmittance and reflectance of the scattered light. It should be additionally noted that T_{Jc}^{IO}=T_{Jc}^{OI} and T_{Ic}^{IO}=T_{Ic}^{OI} for the collimated light.

Equations (S11) and (S12) were used for calculation of intensity of the collimated light transferring from leaf to air across adaxial and abaxial surfaces (J_C^T and I_C^T respectively):

$$J_C^{T} = J_C(0)\cdot(1-F_S)\cdot T_{Jc}^{IO} \quad \text{(S11)}$$

$$I_C^{T} = I_C(h+l)\cdot(1-F_S)\cdot T_{Ic}^{IO} \quad \text{(S12)}$$

Equations (S13) and (S14) were used for calculation of intensity of the collimated light reflecting from adaxial and abaxial leaf surfaces in the lamina (I_C^{RI} and J_C^{RI}, respectively):

$$I_C^{RI} = J_C(0)\cdot(1-F_S)\cdot\left(1-T_{Jc}^{IO}\right) \quad \text{(S13)}$$

$$J_C^{RI} = I_C(h+l)\cdot(1-F_S)\cdot\left(1-T_{Ic}^{IO}\right) \quad \text{(S14)}$$

Transmittance coefficients for the scattered light transfer were described by equations (S15)-(S18). It should be noted that the collimated light was transformed into the scattered light on rough surfaces.

$$T_{Is}^{OI} = \frac{2}{\pi}\int_0^{\pi/2} T_{Ic}^{OI}\, d\beta_{O1} \quad \text{(S15)}$$

$$T_{Js}^{OI} = \frac{2}{\pi}\int_0^{\pi/2} T_{Jc}^{OI}\, d\beta_{O2} \quad \text{(S16)}$$

$$T_{Is}^{IO} = \frac{2}{\pi}\int_0^{\pi/2} T_{Ic}^{IO}\, d\beta_{I1} \quad \text{(S17)}$$

$$T_{Js}^{IO} = \frac{2}{\pi}\int_0^{\pi/2} T_{Jc}^{IO}\, d\beta_{I2} \quad \text{(S18)}$$

where T_{Is}^{OI} and T_{Js}^{OI} are transmittance coefficients for the scattered light transfer from air to leaf on adaxial and abaxial surfaces, respectively; T_{Is}^{IO} and T_{Js}^{IO} are transmittance coefficients for the scattered light transfer from leaf to air on abaxial and adaxial surfaces, respectively. It should be noted that T_{Is}^{OI}= T_{Js}^{OI} и T_{Is}^{IO}= T_{Js}^{IO}; thus, $T_s^{OI,}$ equaling to T_{Is}^{OI} (= T_{Js}^{OI}) and T_s^{IO} equaling to T_{Is}^{IO} (= T_{Js}^{IO}) were used in the analysis. On basis of numerical calculation, it was shown that T_s^{OI}≈0.866 and T_s^{IO}≈0.469. These values were used for modeling.

Equations (S19) and (S20) were used for calculation of intensity of the scattered light transferring from air to leaf across adaxial and abaxial surfaces ($I_S(0)$ and $J_S(h+l)$, respectively):

$$I_S(0) = I_0 \cdot F_S \cdot T_s^{OI} \quad \text{(S19)}$$

$$J_S(h+l) = J_0 \cdot F_S \cdot T_s^{OI} \quad \text{(S20)}$$

Equations (S21) and (S22) were used for calculation of intensity of the scattered light reflecting from adaxial and abaxial leaf surfaces in air (J_S^{RO} and I_S^{RO}, respectively):

$$J_S^{RO} = I_0 \cdot F_S \cdot \left(1-T_s^{OI}\right) \quad \text{(S21)}$$

$$I_S^{RO} = J_0 \cdot F_S \cdot \left(1-T_s^{OI}\right) \quad \text{(S22)}$$

Equations (S23) and (S24) were used for calculation of intensity of the scattered light transferring from leaf to air across adaxial and abaxial surfaces (J_S^T and I_S^T, respectively):

$$J_S^{\ T} = J_C(0)\cdot F_S \cdot T_s^{IO} + J_S(0)\cdot T_s^{IO} \tag{S23}$$

$$I_S^{\ T} = I_C(h+l)\cdot F_S \cdot T_s^{IO} + I_S(h+l)\cdot T_s^{IO} \tag{S24}$$

Equations (S25) and (S26) were used for calculation of intensity of the scattered light reflecting from adaxial and abaxial leaf surfaces in the lamina (I_S^{RI} and J_S^{RI}, respectively):

$$I_S^{\ RI} = J_C(0)\cdot F_S \cdot \left(1-T_s^{IO}\right) + J_S(0)\cdot \left(1-T_s^{IO}\right) \tag{S25}$$

$$J_S^{\ RI} = I_C(h+l)\cdot F_S \cdot \left(1-T_s^{IO}\right) + I_S(h+l)\cdot \left(1-T_s^{IO}\right) \tag{S26}$$

Equations (S27) and (S28) were used for calculation of total intensities of light flows from adaxial and abaxial leaf surfaces to air (J_{out}^1 and I_{out}^1, respectively):

$$J_{out}^{\ 1} = J_C^{\ RO} + J_C^{\ T} + J_S^{\ RO} + J_S^{\ T} \tag{S27}$$

$$I_{out}^{\ 1} = I_C^{\ RO} + I_C^{\ T} + I_S^{\ RO} + I_S^{\ T} \tag{S28}$$

where "1" shows that these light intensities were calculated on basis of the first iteration of the light propagation through leaf.

Equations describing light transmittance in the palisade mesophyll layer

We used Beer–Bouguer–Lambert law as the basis of the description of the light propagation through the palisade mesophyll layer. Equations (S29)-(S32) were used for this description:

$$I_C(x) = I_C(0)\cdot e^{-\frac{a_P}{\cos(\beta_{I1})}x} \tag{S29}$$

$$J_C(x) = J_C(h)\cdot e^{-\frac{a_P}{\cos(\beta_{I2})}(h-x)} \tag{S30}$$

$$I_S(x) = I_S(0)\cdot e^{-2a_P x} \tag{S31}$$

$$J_S(x) = J_S(h)\cdot e^{-2a_P(h-x)} \tag{S32}$$

where x is the coordinate, a_P is the light absorption coefficient, and "2" is the coefficient showing increase of the light absorption (and scattering) for the scattered light (on the basis of pathlength averaging over a hemisphere).

Based on these equations, equations (S33) – (S36), which described the light intensity on borders of the palisade mesophyll layer, were derived:

$$I_C(h) = I_C(0) e^{-\frac{a_P}{\cos(\beta_{I1})}h} \tag{S33}$$

$$J_C(0) = J_C(h)\cdot e^{-\frac{a_P}{\cos(\beta_{I2})}h} \tag{S34}$$

$$I_S(h) = I_S(0)\cdot e^{-2a_P h} \tag{S35}$$

$$J_S(0) = J_S(h)\cdot e^{-2a_P h} + J_S^{\ Add} \tag{S36}$$

where $J_C(0)$ and $I_C(h)$ are intensities of collimated backward and forward light on upper and lower borders of the palisade mesophyll layer, $J_S(0)$ and $I_S(h)$ are intensities of scattered backward and forward light on upper and lower borders of the palisade mesophyll layer, J_S^{Add} is the additional scattered light. J_S^{Add} is caused by the scattering (and changing light direction) of the scattered ($I_S(x)$) and collimated ($I_C(x)$) forward light in the palisade mesophyll layer. The J_S^{Add} should have low intensity; however, it can be important for the plant remote sensing based on measuring reflectance at red and light spectral bands which have high light absorption.

We used equation (S37) to calculate J_S^{Add}:

$$J_S^{\ Add} = I_C(0)\cdot \frac{s_P(1-f)}{\cos(\beta_{I1})}\int_0^h e^{-\frac{a_P}{\cos(\beta_{I1})}x}\cdot e^{-2a_P x}dx + 2I_S(0)\cdot s_P(1-f)\int_0^h e^{-2a_P x}\cdot e^{-2a_P x}dx \tag{S37}$$

where s_P is the light scattering coefficient in the palisade mesophyll layer, f is the asymmetry factor which can describe the anisotropy of scattering (we assumed that $f = 0.5$; i.e., the asymmetry was absent). Equation (S38) is the solution of the Equation (S37):

$$J_S^{Add} = I_C(0) \cdot \frac{s_P(1-f)}{a_P(1+2\cos(\beta_{I1}))} \cdot \left(1 - e^{-\left(\frac{a_P}{\cos(\beta_{I1})} + 2a_P\right)h}\right) + I_S(0) \cdot \frac{s_P(1-f)}{2a_P} \cdot \left(1 - e^{-4a_P h}\right) \quad \text{(S38)}$$

Equations describing light transmittance and scattering in the spongy mesophyll layer

We used the Kubelka-Munk model with four light flows to describe optical properties the spongy mesophyll layer. We used modified coordinate x_1 ($x_1 = x - h$) to simplify the analysis. x_1 can be used from h to $h+l$, only, where h and l are the thicknesses of the palisade and spongy mesophyll layers, respectively.

System of equations (S39) shows the initial Kubelka-Munk equations:

$$\begin{aligned}
\frac{dI_C(x_1)}{dx_1} &= -\frac{a_{Sp} + s_{Sp}}{\cos(\beta_{I1})} \cdot I_C(x_1) \\
\frac{dJ_C(x_1)}{dx_1} &= \frac{a_{Sp} + s_{Sp}}{\cos(\beta_{I2})} \cdot J_C(x_1) \\
\frac{dI_S(x_1)}{dx_1} &= f \cdot \frac{s_{Sp}}{\cos(\beta_{I1})} \cdot I_C(x_1) + (1-f) \cdot \frac{s_{Sp}}{\cos(\beta_{I2})} \cdot J_C(x_1) - \\
&- 2\left(a_{Sp} + s_{Sp} \cdot (1-f)\right) \cdot I_S(x_1) + 2s_{Sp} \cdot (1-f) \cdot J_S(x_1) \\
\frac{dJ_S(x_1)}{dx_1} &= -(1-f) \cdot \frac{s_{Sp}}{\cos(\beta_{I1})} \cdot I_C(x_1) - f \cdot \frac{s_{Sp}}{\cos(\beta_{I2})} \cdot J_C(x_1) - \\
&- 2s_{Sp} \cdot (1-f) \cdot I_S(x_1) + 2\left(a_{Sp} + s_{Sp} \cdot (1-f)\right) \cdot J_S(x_1)
\end{aligned} \quad \text{(S39)}$$

where a_{Sp} and s_{Sp} are light absorption and scattering coefficients in the spongy mesophyll.

System of equations (S39) was transformed to the system of equations (S40) to simplify analysis:

$$\begin{aligned}
\frac{dI_C(x_1)}{dx_1} &= L_{11} \cdot I_C(x_1) \\
\frac{dJ_C(x_1)}{dx_1} &= L_{22} \cdot J_C(x_1) \\
\frac{dI_S(x_1)}{dx_1} &= L_{31} \cdot I_C(x_1) + L_{32} \cdot J_C(x_1) + L_{33} \cdot I_S(x_1) + L_{34} \cdot J_S(x_1) \\
\frac{dJ_S(x_1)}{dx_1} &= L_{41} \cdot I_C(x_1) + L_{42} \cdot J_C(x_1) + L_{43} \cdot I_S(x_1) + L_{44} \cdot J_S(x_1)
\end{aligned} \quad \text{(S40)}$$

where coefficients (L) correspond to coefficients of light flows in the system of equations (S39).

We used the method of undetermined coefficients describing light flows as the combination of elementary exponents ($I_C(x) = Ae^{\lambda x_1}$, $J_C(x) = Be^{\lambda x_1}$, $I_S(x) = Ce^{\lambda x_1}$, and $J_S(x) = De^{\lambda x_1}$)

Equation (S41) is the characteristic equation of this system:

$$(L_{11} - \lambda) \cdot (L_{22} - \lambda) \cdot \left[(L_{33} - \lambda) \cdot (L_{44} - \lambda) - L_{34} L_{43}\right] = 0 \quad \text{(S41)}$$

Solution of this equation is equations (S42):

$$\begin{aligned}
\lambda_1 &= -L_{11} \\
\lambda_2 &= -L_{22} \\
\lambda_3 &= \frac{L_{33} + L_{44}}{2} + \sqrt{\left(\frac{L_{33} + L_{44}}{2}\right)^2 - \left(L_{33} \cdot L_{44} - L_{34} \cdot L_{43}\right)} \\
\lambda_4 &= \frac{L_{33} + L_{44}}{2} - \sqrt{\left(\frac{L_{33} + L_{44}}{2}\right)^2 - \left(L_{33} \cdot L_{44} - L_{34} \cdot L_{43}\right)}
\end{aligned} \quad \text{(S42)}$$

Thus, system of equations (S43) was used to describe optical properties of the spongy mesophyll layer (for x ranging from h to $h+l$):

$$I_C(x) = A_1 e^{\lambda_1 (x-h)} + A_2 e^{\lambda_2 (x-h)} + A_3 e^{\lambda_3 (x-h)} + A_4 e^{\lambda_4 (x-h)}$$

$$J_C(x) = B_1 e^{\lambda_1(x\text{-}h)} + B_2 e^{\lambda_2(x\text{-}h)} + B_3 e^{\lambda_3(x\text{-}h)} + B_4 e^{\lambda_4(x\text{-}h)} \quad \text{(S43)}$$
$$I_S(x) = C_1 e^{\lambda_1(x\text{-}h)} + C_2 e^{\lambda_2(x\text{-}h)} + C_3 e^{\lambda_3(x\text{-}h)} + C_4 e^{\lambda_4(x\text{-}h)}$$
$$J_S(x) = D_1 e^{\lambda_1(x\text{-}h)} + D_2 e^{\lambda_2(x\text{-}h)} + D_3 e^{\lambda_3(x\text{-}h)} + D_4 e^{\lambda_4(x\text{-}h)}$$

where A_1, A_2, A_3, A_4, B_1, B_2, B_3, B_4, C_1, C_2, C_3, C_4, D_1, D_2, D_3, and D_4 are constants.

System of equations (S44) was used for description of boundary conditions:

$$A_1 + A_2 + A_3 + A_4 = I_C(h)$$
$$B_1 e^{\lambda_1 l} + B_2 e^{\lambda_2 l} + B_3 e^{\lambda_3 l} + B_4 e^{\lambda_4 l} = J_C(h+l) \quad \text{(S44)}$$
$$C_1 + C_2 + C_3 + C_4 = I_S(h)$$
$$D_1 e^{\lambda_1 l} + D_2 e^{\lambda_2 l} + D_3 e^{\lambda_3 l} + D_4 e^{\lambda_4 l} = J_S(h+l)$$

$I_C(x)$ and $J_C(x)$ cannot be dependent on other light flows. It means that $A_2=A_3=A_4=0$ and $B_1=B_3=B_4=0$; in contrast, $A_1=I_C(h)$ and $B_2=J_C(h+l)$.

Based on elementary exponents and equations (S40) for $I_S(x)$ and $J_S(x)$, the system of equation (S45) was derived:

$$C \cdot (L_{33} - \lambda) + D \cdot L_{34} = -(A \cdot L_{31} + B \cdot L_{32}) \quad \text{(S45)}$$
$$C \cdot L_{43} + D \cdot (L_{44} - \lambda) = -(A \cdot L_{41} + B \cdot L_{42})$$

Equations (S46) and (S47) are solutions of this system at λ_1 ($A_1=I_C(h)$ and $B_1=0$):

$$C_1 = I_C(h) \cdot \frac{\text{-}L_{31} \cdot (L_{44} - \lambda_1) + L_{41} \cdot L_{34}}{(L_{33} - \lambda_1) \cdot (L_{44} - \lambda_1) - L_{34} \cdot L_{43}} \quad \text{(S46)}$$

$$D_1 = I_C(h) \cdot \frac{\text{-}L_{41} \cdot (L_{33} - \lambda_1) + L_{31} \cdot L_{43}}{(L_{33} - \lambda_1) \cdot (L_{44} - \lambda_1) - L_{34} \cdot L_{43}} \quad \text{(S47)}$$

Equations (S48) and (S49) are solutions of this system at λ_2 ($A_2=0$ and $B_2 = J_C(h+l) \cdot e^{-\lambda_2 l}$):

$$C_2 = J_C(h+l) \cdot e^{-\lambda_2 l} \cdot \frac{\text{-}L_{32} \cdot (L_{44} - \lambda_2) + L_{42} \cdot L_{34}}{(L_{33} - \lambda_2) \cdot (L_{44} - \lambda_2) - L_{34} \cdot L_{43}} \quad \text{(S48)}$$

$$D_2 = J_C(h+l) \cdot e^{-\lambda_2 l} \cdot \frac{\text{-}L_{42} \cdot (L_{33} - \lambda_2) + L_{32} \cdot L_{43}}{(L_{33} - \lambda_2) \cdot (L_{44} - \lambda_2) - L_{34} \cdot L_{43}} \quad \text{(S49)}$$

Equations (S50) and (S51) describe relations between C_3 and D_3 for λ_3 and C_4 and D_4 for λ_4:

$$D_3 = -C_3 \cdot \frac{L_{33} - \lambda_3}{L_{34}} \quad \text{(S50)}$$

$$D_4 = -C_4 \cdot \frac{L_{33} - \lambda_4}{L_{34}} \quad \text{(S51)}$$

Based on the system of equations (S44), the system of equations (S52) was derived:

$$C_3 + C_4 = I_S(h) - C_1 - C_2 \quad \text{(S52)}$$
$$D_3 e^{\lambda_3 l} + D_4 e^{\lambda_4 l} = J_S(h+l) - D_1 e^{\lambda_1 l} - D_2 e^{\lambda_2 l}$$

where C_1, C_2, D_1, and D_2 can be calculated with equations (S46)-(S49). Combining equations (S50), (S51), and (S52), we derived the system of equations (S53):

$$C_3 + C_4 = I_S(h) - C_1 - C_2 \quad \text{(S53)}$$
$$C_3 \frac{L_{33} - \lambda_3}{L_{34}} e^{\lambda_3 l} + C_4 \frac{L_{33} - \lambda_4}{L_{34}} e^{\lambda_4 l} = -J_S(h+l) + D_1 e^{\lambda_1 l} + D_2 e^{\lambda_2 l}$$

Equations (S54) and (S55) are solutions of this system:

$$C_3 = \frac{(I_S(h) - C_1 - C_2) \cdot (L_{33} - \lambda_4) e^{\lambda_4 l} - \left(-J_S(h+l) + D_1 e^{\lambda_1 l} + D_2 e^{\lambda_2 l}\right) \cdot L_{34}}{(L_{33} - \lambda_4) e^{\lambda_4 l} - (L_{33} - \lambda_3) e^{\lambda_3 l}} \quad \text{(S54)}$$

$$C_4 = \frac{\left(-J_S(h+l) + D_1 e^{\lambda_1 l} + D_2 e^{\lambda_2 l}\right) \cdot L_{34} - (I_S(h) - C_1 - C_2) \cdot (L_{33} - \lambda_3) e^{\lambda_3 l}}{(L_{33} - \lambda_4) e^{\lambda_4 l} - (L_{33} - \lambda_3) e^{\lambda_3 l}} \quad \text{(S55)}$$

Combining equations (S50), (S51), (S54), and (S55), we derived equations (S56) and (S57) to calculate D_3 and D_4:

$$D_3 = -\frac{L_{33}-\lambda_3}{L_{34}}\frac{\left(I_S(h)-C_1-C_2\right)\cdot\left(L_{33}-\lambda_4\right)e^{\lambda_4 l}-\left(-J_S(h+l)+D_1e^{\lambda_1 l}+D_2e^{\lambda_2 l}\right)\cdot L_{34}}{\left(L_{33}-\lambda_4\right)e^{\lambda_4 l}-\left(L_{33}-\lambda_3\right)e^{\lambda_3 l}} \quad \text{(S56)}$$

$$D_4 = -\frac{L_{33}-\lambda_4}{L_{34}}\frac{\left(-J_S(h+l)+D_1e^{\lambda_1 l}+D_2e^{\lambda_2 l}\right)\cdot L_{34}-\left(I_S(h)-C_1-C_2\right)\cdot\left(L_{33}-\lambda_3\right)e^{\lambda_3 l}}{\left(L_{33}-\lambda_4\right)e^{\lambda_4 l}-\left(L_{33}-\lambda_3\right)e^{\lambda_3 l}} \quad \text{(S57)}$$

Description of several iterations of the light propagation through leaf

Equations from previous sections can be used for the first iteration of calculation of light transmittance and reflectance. However, equations (S13), (S14), (S25), and (S26) show that light can secondarily input into the leaf lamina; i.e. the second iteration of calculation of the light propagation is possible (as well as the third iteration, fourth iterations, fifth iteration, etc.). This effect can be large at low light absorption coefficient (particularly, for the near infrared light, NIR).

The second iteration of calculation of the light propagation through the leaf and light transmittance from lamina to air was based on the same equations, which were used for the first iteration, after substitution of following parameters: $I_C(0)=I_C^{RI}$, $J_C(h+l)=J_C^{RI}$, $I_S(0)=I_S^{RI}$, $J_S(h+l)=J_S^{RI}$, $\beta_{I1}=\beta_{I2}$, and $\beta_{I2}=\beta_{I1}$.

Calculated after this procedure I_C^{RI}, J_C^{RI}, I_S^{RI}, and J_S^{RI} could be used for the third iteration of calculation, etc. Thus, equations (S58) and (S59) could be used for calculation of light outputs from adaxial and abaxial leaf surfaces (J_{out} and I_{out}, respectively):

$$J_{out} = \sum_{i=1}^{N} J_{out}^{\ i} \quad \text{(S58)}$$

$$I_{out} = \sum_{i=1}^{N} I_{out}^{\ i} \quad \text{(S59)}$$

where J_{out}^i and I_{out}^i are light outputs from adaxial and abaxial leaf surfaces, respectively, which are calculated on iteration *i*, *N* is the quantity of the iterations which is necessary to approximately describe the reflectance and transmittance spectra of leaves.

Description of light absorption coefficients

In the current model, we considered that the light absorption coefficient was the function of the light wavelength through photosynthetic pigments and complexes formed by these pigments. Concentration of photosynthetic pigments in the spongy mesophyll layer was 20% from this concentration in the palisade mesophyll layer ($N_{Sp/P}$=0.2):

$$a_{Sp}(\lambda) = N_{Sp/P}\cdot a_P(\lambda) \quad \text{(S60)}$$

where $a_P(\lambda)$ and $a_{Sp}(\lambda)$ are spectra of the light absorption coefficient of layers of the palisade and spongy mesophyll. $a_P(\lambda)$ and $a_{Sp}(\lambda)$ were used as a_P and a_{Sp} in the analysis.

Equation (S61) was used for calculation of $a_P(\lambda)$:

$$a_P(\lambda) = C_{ChA}\cdot a_{ChA}(\lambda) + C_{ChB}\cdot a_{ChB}(\lambda) + C_{Car}\cdot a_{Car}(\lambda) + C_{Anth}\cdot a_{Anth}(\lambda) \quad \text{(S61)}$$

where C_{ChA}, C_{ChB}, C_{Car}, and C_{Anth} are concentrations (mg cm^{-3}) of chlorophyll a, chlorophyll b, carotenoids, and anthocyanin respectively, in the palisade mesophyll, $a_{ChA}(\lambda)$, $a_{ChB}(\lambda)$, $a_{Car}(\lambda)$, and $a_{Anth}(\lambda)$,are spectra of specific light absorption coefficients (cm^2 mg^{-1}) of chlorophyll a, chlorophyll b, carotenoids, and anthocyanin respectively. These spectra are shown in Figure 1b. It should be noted that equation (S61) additionally includes anthocyanin, which was not described in the initial model.

Equation (S62) was used for calculation of the average concentration of each photosynthetic pigment (C^{av}) in leaves:

$$C^{av} = C\cdot\left(\frac{h}{l+h} + N_{Sp/P}\cdot\frac{l}{l+h}\right) \quad \text{(S62)}$$

where *C* is the pigment concentration in the palisade mesophyll layer.
